# Advancements in copy number variation screening in herbivorous livestock genomes and their association with phenotypic traits

**DOI:** 10.3389/fvets.2023.1334434

**Published:** 2024-01-11

**Authors:** Xiaotong Liu, Wenting Chen, Bingjian Huang, Xinrui Wang, Yongdong Peng, Xinhao Zhang, Wenqiong Chai, Muhammad Zahoor Khan, Changfa Wang

**Affiliations:** Liaocheng Research Institute of Donkey High-Efficiency Breeding, Liaocheng University, Liaocheng, China

**Keywords:** copy number variation, herbivorous livestock, phenotypes, genome, molecular markers

## Abstract

Copy number variations (CNVs) have garnered increasing attention within the realm of genetics due to their prevalence in human, animal, and plant genomes. These structural genetic variations have demonstrated associations with a broad spectrum of phenotypic diversity, economic traits, environmental adaptations, epidemics, and other essential aspects of both plants and animals. Furthermore, CNVs exhibit extensive sequence variability and encompass a wide array of genomes. The advancement and maturity of microarray and sequencing technologies have catalyzed a surge in research endeavors pertaining to CNVs. This is particularly prominent in the context of livestock breeding, where molecular markers have gained prominence as a valuable tool in comparison to traditional breeding methods. In light of these developments, a contemporary and comprehensive review of existing studies on CNVs becomes imperative. This review serves the purpose of providing a brief elucidation of the fundamental concepts underlying CNVs, their mutational mechanisms, and the diverse array of detection methods employed to identify these structural variations within genomes. Furthermore, it seeks to systematically analyze the recent advancements and findings within the field of CNV research, specifically within the genomes of herbivorous livestock species, including cattle, sheep, horses, and donkeys. The review also highlighted the role of CNVs in shaping various phenotypic traits including growth traits, reproductive traits, pigmentation and disease resistance etc., in herbivorous livestock. The main goal of this review is to furnish readers with an up-to-date compilation of knowledge regarding CNVs in herbivorous livestock genomes. By integrating the latest research findings and insights, it is anticipated that this review will not only offer pertinent information but also stimulate future investigations into the realm of CNVs in livestock. In doing so, it endeavors to contribute to the enhancement of breeding strategies, genomic selection, and the overall improvement of herbivorous livestock production and resistance to diseases.

## 1 Introduction

China, renowned as one of the earliest nations to engage in livestock domestication (1), has a rich history of nurturing herbivorous livestock, including cattle, sheep, horses, and donkeys. This ancient practice has played a pivotal role in fulfilling diverse human needs, ranging from the procurement of essential animal-derived products such as meat, eggs, milk, and leather to harnessing domesticated animals for laborious tasks (2). Over time, the scope of domestication has expanded to encompass a multitude of applications. Throughout this evolutionary process, natural and artificial selection mechanisms have yielded an array of domestic animal breeds characterized by varying traits (3), including phenotypic attributes, economic characteristics, environmental adaptability, and resistance to diseases. Nonetheless, the intricate genetic underpinnings responsible for these disparities remain incompletely elucidated.

In recent years, the exploration of genomic variation has emerged as a central focus of scientific inquiry in the fields of animal production and health regulation, as evidenced by numerous studies (4–10). This emphasis on genetic variation holds significant significance in our quest to comprehend the intricate interplay between genetic diversity and a wide array of phenotypic and economic traits exhibited by animals (11–13). Furthermore, it serves as a robust theoretical foundation for elucidating genetic mechanisms and advancing the field of molecular breeding. Since the introduction of genomic selection, a range of livestock species, including sheep, goats, cattle, and horses, have undergone genotyping to assess their suitability for important economic traits, as demonstrated by previous studies (14–16). Up to this point, single nucleotide polymorphisms (SNPs) have been the primary focus of genomic research within the animal breeding community (17, 18). Significant strides have been made in establishing a solid genetic foundation for enhancing production and disease resistance in animals (18, 19). However, it is worth noting that despite these advancements, ~25% of the identified copy number variants (CNVs) exhibit no significant linkage disequilibrium with any SNP, leading to the conclusion that CNVs harbor genetic information that cannot be solely elucidated through SNP analysis (20).

CNVs are heritable chromosomal structural variations, characterized by deletions or insertions exceeding 50 base pairs (21). Notably, CNVs encompass a larger proportion of the genome compared to SNPs (22, 23). Consequently, CNVs are being proposed as an additional reservoir of information to elucidate the genetic variance underlying complex traits that may not be fully accounted for by SNPs alone (20). To date, several methodologies have been commonly employed for CNV detection, including comparative genome hybridization, extracting CNV data from SNP arrays, and whole-genome sequencing (WGS) approaches (24–26). Notably, recent research endeavors have delved into the investigation of the association between CNV in specific genes and a variety of phenotypic traits in animals, including growth characteristics in cattle (27, 28), goats (29–31), sheep (32) and horses (33–38). These studies have also extended their focus to examine the link between CNVs and other vital phenotypes, such as reproduction traits (39, 40) and disease resistance (7, 41). These studies have unveiled CNV as a key player linked to diverse facets of phenotypic diversity and economic traits in animal realms.

**Figure 1:**
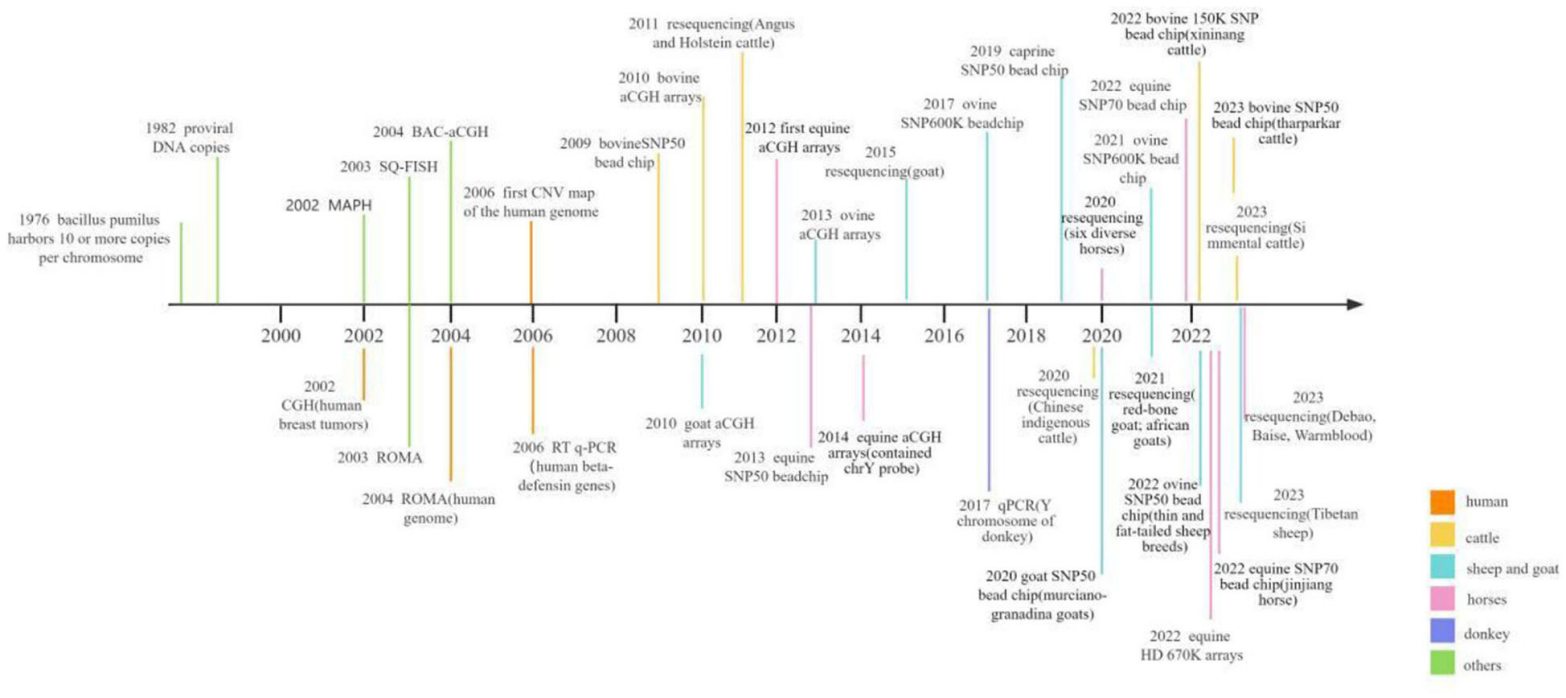
Graphical presentation of the research progress on CNV screening in herbivorous livestock and humans.

The confluence of two pivotal trends, the rising prevalence of molecular markers in livestock breeding and the maturation of microarray and sequencing technologies, necessitates a contemporary and comprehensive review of the burgeoning body of research on CNVs. This review paper seeks to illuminate the intrinsic value and biological ramifications of CNV in the landscape of genetic variation, with a particular focus on its potential as a potent molecular marker in the realm of livestock breeding. Through this approach, our review aims to introduce fresh viewpoints regarding genetic diversity and molecular breeding. Thus, in current review, we have focused on the progress of CNV screening methods in genomes of various herbivorous livestock including cattle, horses, donkeys, sheep and goat. In addition, we have briefly evaluated the association of CNVs in genes with different phenotypic traits including growth traits, reproductive traits, pigmentation, disease resistance, and environmental adaptability, etc., in cattle, horses, sheep and goat. The overall progress in screening CNVs within livestock genomes is summarized in Figure 1 (32–35, 37, 42–70).

## 2 Overview of CNV biology

### 2.1 CNV definition

**Figure 2:**
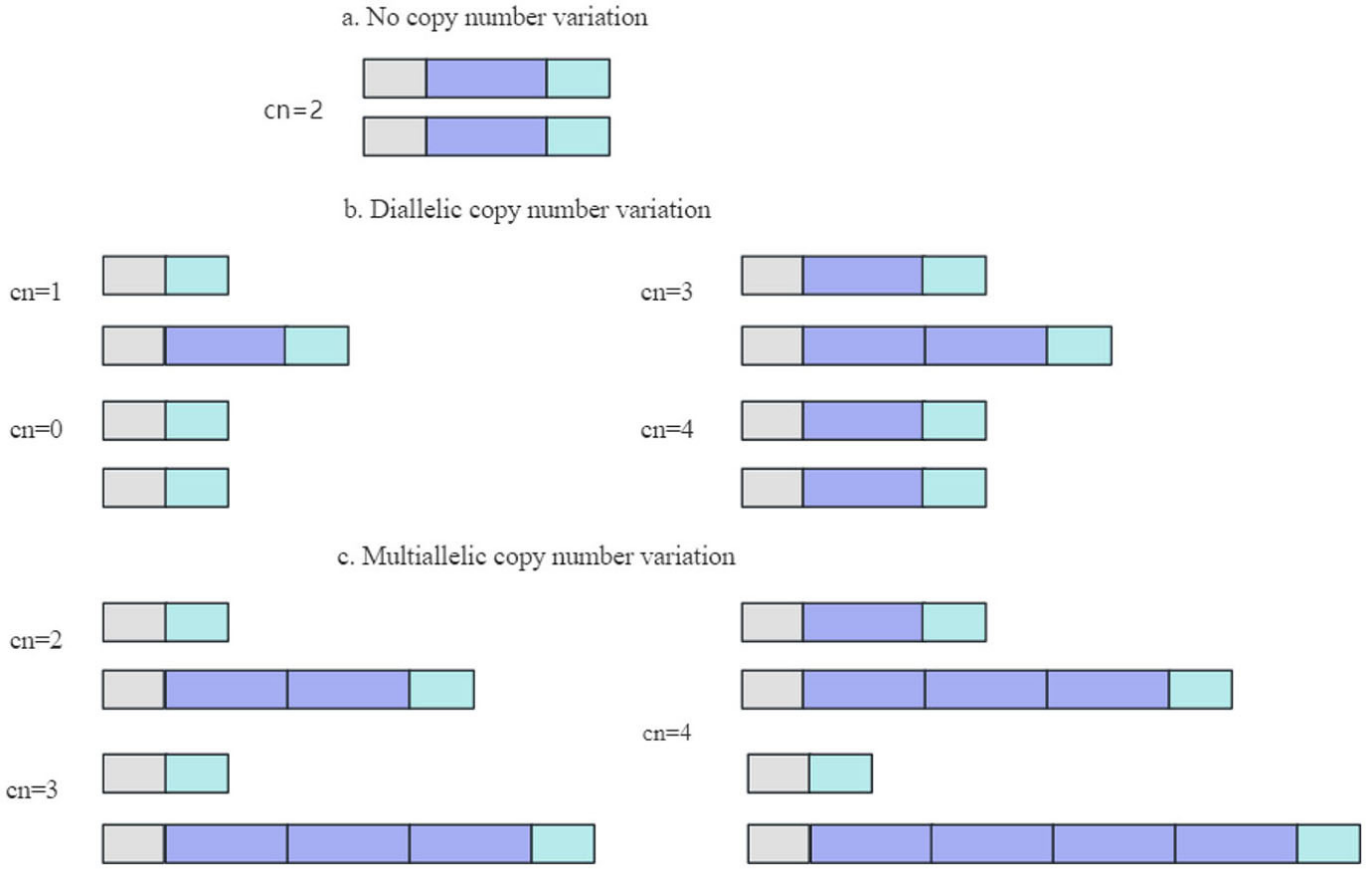
Common types of CNV.

A CNV, typically resulting from genome rearrangement, refers to the amplification or reduction in the copy number of a large genome segment of 1 kb or greater in length, primarily demonstrated via sub-microscopic deletions and duplications (50, 71). The common variant forms of CNV are illustrated in Figure 2 (72).

Several nearby CNVs and partially overlapping CNVs in the same genomic region can be merged into a single CNV segment (73). CNVs play a vital role in genomic structural variation (SV) (74). The number of base pairs regulated by CNVs is over five times greater than the number regulated by single nucleotide variants (SNVs) in each individual. Each CNV is correlated more than three times with one genome-wide association signal and fifty times with expression quantitative trait loci (eQTL) compared to SNVs (75). Furthermore, CNVs have been recognized for its significant influence on the evolution of phenotypic diversity, disease resistance, and evolutionary processes in organisms (76, 77).

It is widely recognized that CNVs are common in the genomes of plants, animals, and humans (78), but there is no final conclusion on the mechanism of CNV formation. According to existing studies, three primary formation mechanisms may be involved: (1) Non-allelic homologous Recombination, NAHR: Rearrangements occurring between homologous chromosomes of an individual's genomic DNA during meiosis can lead to duplications, deletions, inversions, and translocations (79, 80). (2) Non-homologous end-joining, NHEJ: A mechanism of genomic rearrangement that occurs during the period of DNA double-strand break repair and that can result in numerous simple CNVs (79, 80). (3) Replication fork smiling and template Switching, FoSTeS: Refers to the stalling of DNA replication forks which causes the lagging strand to break away from the DNA template and switch to other replication forks to continue DNA replication synthesis. This can lead to DNA duplications or deletions and the emergence of a significant number of CNVs. Consequently, the formation of complex structural CNVs may be attributed to this phenomenon (80, 81).

### 2.2 CNV detection methods

#### 2.2.1 Chip technology

Array comparative genomic hybridization (aCGH) has proven to be an efficient technique for the identification of CNVs at a genome-wide level (82–84). The fundamental principle of CGH involves labeling the DNA under analysis and control DNA with distinct fluorescent dyes. Subsequently, these treated DNAs are hybridized to standard chromosomes, and a digital fluorescence imaging system is employed to scan them, with a priority on fluorescence intensity ratios (85, 86). The aCGH is a microarray technology that utilizes two types of microarrays depending on the probes used during fabrication: Bacterial Artificial Chromosome CGH microarrays and Oligonucleotide Probe CGH microarrays. These aCGH microarray probes cover the entire genome and exhibit exceptional sensitivity, accuracy, and resolution, facilitating high-throughput screening. Previous research has provided robust support for the credibility of the experimental data generated by CGH (87, 88).

Single nucleotide polymorphism (SNP) microarray technology represents another effective method for detecting CNVs. SNP microarray technology requires only a single hybridization, thus obviating the need for the simultaneous double hybridization of two DNA samples with probes. It determines the genomic copy equivalents of each locus by comparing variations in signal intensities between the samples being tested (89). A SNP microarray demonstrates remarkable stability and high resolution, enabling the detection of diverse forms of CNVs, including sub-microscopic deletions, duplications, and more (90). In comparison to Comparative Genomic Hybridization, SNP microarrays offer the advantage of simultaneously detecting CNVs while determining their genotypes and revealing heterozygous deletions. This approach is not only more cost-effective but also facilitates large-scale CNV testing (91). Several software and programs are currently available for CNV detection using chip technology, including CNVPartition, PennCNV, and QuantiSNP. A study revealed through genome sequencing of horses that the optimal order of performance for the three assays was CNVPartition, PennCNV, and QuantiSNP (92). Furthermore, the combination of PennCNV and QuantiSNP exhibited improved accuracy in CNV detection.

#### 2.2.2 Sequencing technology

As gene sequencing technology has matured, numerous tools and software have been developed to enhance the efficiency and precision of copy number variant detection. Next-generation sequencing (NGS) has emerged as the most commonly used method for detecting CNVs in recent years (93), with Illumina's Solexa/HiSeq technology being a prominent representative. Second-generation sequencing technology aims to synthesize and sequence DNA simultaneously. Fluorescent signals are excited by lasers and recorded using optical equipment. These recorded signals are then converted into bases using computer technology. Second-generation sequencing methods are not limited to target sequences hybridized with primer probes and can identify genome-wide CNVs, structural variants, and other variations (94). In the current landscape, four primary strategies and methods are employed for detecting variants in NGS data: paired-end mapping (PEM), split-read (SR), read depth (RD), and *de novo* assembly (AS), in addition to a combined approach based on the above four methods. Compared to microarray-based methods, next-generation sequencing technology offers advantages in terms of speed, resolution, cost-effectiveness, and reproducibility (95–97). Furthermore, widely used sequencing technologies for identifying CNVs, software including CNVnator, CNVpytor, CNVcaller, and ERDS have been documented.

## 3 Overview of screening methods for CNVs in herbivorous livestock genomes

### 3.1 Cattle genome

Cattle ranching represent a promising industry with substantial economic, ecological, and social implications. The diversity of cattle breeds, including Holstein for dairy production, Charolais for beef, and regionally adapted yaks and buffaloes, caters to a wide range of human needs. CNVs assume significant importance in the exploration of phenotypic traits and adaptation to various environments, shedding light on the domestication origins of these animals. As dietary preferences evolve toward beef with higher protein and lower fat content, copy number variation regions (CNVRs) have been found to be associated with cattle carcass traits, offering theoretical support for future breeding endeavors. In recent years, numerous investigations have identified CNVs within the bovine genome. Table 1 provides a comprehensive summary of some of these studies. Consistently, a study identified a total of 79 CNV loci across six distinct cattle breeds, employing the BovineSNP50 BeadChip (51). Notably, 10 CNVs were found to overlap with those previously identified through aCGH data. Similarly another study conducted an extensive genome-wide analysis of CNVs in 90 modern domesticated cattle, uncovering over 200 potential CNVRs (52). In addition, this high-quality bovine CNV map fills critical gaps in current genome-wide association and selection studies based on SNP genotyping. Previous studies generated genome-wide CNV maps for cattle using the BovineSNP50 BeadChip and aCGH arrays, respectively (22, 98). Similarly, Kumar et al. (69) pioneered CNV detection in Indian Tharparkar cattle, identifying a total of 8881 CNVs, which were subsequently filtered down to 693 CNVs and merged into 447 CNVRs, representing ~2.17% of the cattle genome. Utilizing a purebred Angus cow as a reference, a study documented 605 CNVRs through a genome-wide analysis of CNVs in comparative genomic crossbreeding arrays of 29 Chinese domestic bulls (100). Detailed distribution maps of these CNVRs were constructed on their respective genomes. In a consistent manner, Kooverjee et al. (103) conducted a comprehensive investigation, wherein they successfully identified a total of 355 CNVRs in a cohort of five crossbred cows. This identification was accomplished through the utilization of the Panelcn. MOPS software. Notably, these CNVRs exhibited an average length of 9318 base pairs, collectively representing ~0.15% coverage of the bovine genome. Previous studies employed different platforms for genotyping Holstein cattle, followed by CNV analysis, to investigate the impact of genotype array density on CNV detection, thereby contributing to our understanding of genetic variation in Holstein cattle (24, 26). Accordingly, Sun et al. (104) harnessed sequencing technology to sequence the entire genome of Simmental bulls, detecting 2944 CNVRs, which were subsequently subject to genetic analysis, revealing associations with reproduction, immunity, and fertility. These findings constitute a valuable molecular breeding resource for cattle. A study analyzed common CNV regions in Xinjiang brown cattle and compared differences between the ARS and UMD reference genomes, suggesting the ARS reference genome's superior effectiveness in CNV detection (102). Likewise, previously studies conducted on CNVs in the bovine genome utilizing the BovineHD Genotyping BeadChip (38, 102). Conversely, studies undertook an analysis of CNVs in the bovine genome through sequencing technologies (23, 63, 99, 105).

**Table 1:** Summary of CNVs and identification methods performed in cattle.

**References**	**Methods**	**Species**	**Samples**	**CNV number**	**CNVR number**
Matukumalli et al. (51)	BovineSNP50 BeadChip	6	576	79	-
Liu et al. (52)	Bovine aCGH arrays	3	90	1041	229
Bae et al. (98)	BovineSNP50 BeadChip	1	265	885	368
Fadista et al. (22)	Bovine aCGH arrays	4	20	-	304
Stothard et al. (23)	Resequencing	2	2	790	-
Bickhart et al. (99)	Resequencing	3	6	1265	-
Zhang et al. (100)	Bovine aCGH arrays	15	29	-	605
Da Silva et al. (101)	Bovine HD Genotyping Bead Chip, Resequencing	1	1717	68007	7319
Zhou et al. (102)	BovineHD Genotyping BeadChip	1	528	191	-
Liu et al. (60)	Resequencing	1	14	-	1344
Mei et al. (63)	Resequencing	8	75	-	11486
Butty et al. (26)	BovineHD Beadchip, Genome Profiler Bovine 150 K, Genome Profiler Bovine HD, BovineSNP50 Beadchip	1	96	-	52/36
Butty et al. (24)	BovineHD Beadchip, Genome Profiler Bovine 150K, Genome Profiler Bovine HD, BovineSNP50 Beadchip, Genome Profiler Bovine 50K	1	5845	23256	1645
Zhou et al. (67)	Bovine 150K SNP BeadChip	1	403	-	38/33
Kooverjee et al. (103)	Resequencing	3	5	-	355
Kumar et al. (69)	BovineSNP50 BeadChip	1	72	693	447
Sun et al. (104)	Resequencing	1	30	-	2944

### 3.2 Sheep and goat genomes

Sheep products, encompassing mutton meat, milk, wool, and cashmere, are of considerable significance within the sheep industry. They not only enhance the quality of life for individuals but also contribute substantially to industrial development, augment the incomes of farmers and herdsmen, and provide high-quality fertilizers for farmland, among other advantages. Genomic selection technology has emerged as a pivotal approach in sheep breeding. Current research has unveiled associations between CNVs and various traits in sheep, including growth characteristics, wool color, cashmere quality, disease resistance, and reproduction.

Numerous studies focused on mapping CNVs within the sheep genome have significantly enriched our understanding of genomic variations in sheep. Table 2 provides a comprehensive summary of research pertaining to CNVs in the sheep genome. A study identified a total of 238 CNVRs and establishing a CNV map in the genomes of three distinct sheep breeds using the sheep SNP50K microarray (54). Consequently, Ma et al. (106) investigated CNVs in the genomes of eight sheep breeds using the sheep SNP50K microarray, identifying 111 CNV regions from 160 sheep and mapping the distribution of CNVRs across autosomal chromosomes. Consistently, total of 13,347 CNVs based on sequencing data from six domesticated goats and two wild goats were detected (56). While another experimental trial employed high-density sheep SNP microarrays to identify 371, 301, and 66 autosomal CNVRs within the genomes of big-tailed frigid sheep, Altai sheep, and Tibetan sheep, respectively (107). This endeavor resulted in the creation of the first high-resolution sheep CNV map, offering a valuable resource for comprehending genomic variation in sheep. In continuity, Yang et al. (108) identified 24,558 CNVs from 2,254 sheep across various geographic regions worldwide, culminating in 619 CNV regions with a combined length of 197 megabases (Mb). This encompasses 6.9% of the sheep genome and establishes a comprehensive CNV map that can assist in genome annotation for sheep. In addition, Igoshin et al. (111) detected 4,527 CNVs among 354 sheep representing 16 Russian indigenous breeds. Gene function enrichment analysis revealed significant impacts of CNVs on olfactory perception and immunity within 12 of the breeds. In line, Taghizadeh et al. (68) identified 328 and 187 CNVRs in fat-tailed and thin-tailed sheep breeds, respectively. These CNVRs were found to be located within or overlapping with 790 known sheep genes, covering ~73.85 Mb of the sheep genome. Previous report conducted a genome-wide analysis on 48 beach sheep, documented 1,296 CNV regions and constructed a CNVs map of the Tan sheep genome, thereby complemented the data on CNVs in the Chinese sheep genome (59). While another study identified 6,286 CNVs in a total of 1,023 sheep representing 50 different breeds worldwide, employing the pennCNV tool. The results unveiled differences in CNVs among populations across different geographic regions (60). Furthermore, a study reported 42 CNVs from 120 samples representing five dairy goat breeds and established significant associations between two CNVs (CNV5 and CNV25) and two milk production traits (109). Consequently, Guan et al. (62) detected 1,461 regions of CNV within the Spanish dairy goat genome, with an average length of 196.89 kilobases (kb). The total length of all CNV regions accounted for 3.9% of the autosomal genome, leading to the creation of a CNV map. Previous study has identified 127 CNVRs in four breeds of goats, covering ~11.39 Mb of the bovine genome, thereby establishing the first CNVR map for goats (70). In addition, a study reported 1,217 CNVRs in 67 sheep breeds worldwide (66). Furthermore, a study identified 4,769 high-quality CNVRs in 47 sheep breeds globally, subsequently generating CNV maps. Additionally, they investigated the influence of solar radiation on CNVs within sheep genomes (66). Consistently, a study employed resequencing to detect CNVs in two Tibetan sheep breeds, ultimately identifying 368 distinct CNVRs, which may contribute to determining population disparities (32). Yuan and co-authors were the first to utilize resequencing technology to establish a CNV map of Chinese fine-wool sheep and analyzed the overlap of CNVRs with several quantitative trait loci related to economic traits, providing vital insights for the future improvement of fine-wool sheep (110). Consistently previous studies employed resequencing to analyze the genomic CNVs of Mile red-boned goats and African goats, respectively (64, 65). These findings facilitated a deeper understanding of the genetic traits of these animals. Consequently, another study identified a total of 702 CNVs in 120 dairy goats, resulting in the creation of a CNVR map, which promises to be beneficial for further research on the association between CNVs and phenotypic variations (13).

**Table 2:** Summary of CNVs and identification methods performed in sheep and goat.

**References**	**Methods**	**Species**	**Samples**	**CNV number**	**CNVR number**
Fontanesi et al. (70)	aCGH	4	9	161	127
Liu et al. (54)	Ovine SNP50 BeadChip	3	50	3624	238
Ma et al. (106)	Ovine SNP50 BeadChip	8	160	173	111
Dong et al. (56)	Resequencing	2	8	13347	-
Zhu et al. (107)	Ovine HD 600K SNP arrays	3	110	-	738
Ma et al. (59)	Ovine SNP600K BeadChip	1	48	5190	1296
Yang et al. (108)	Ovine SNP50 BeadChip	68	2111	24588	619
Liu et al. (105)	CaprineSNP50 BeadChip	50	1023	6286	978
Kang et al. (109)	CaprineSNP50 BeadChip	5	120	42	-
Guan et al. (62)	Goat SNP50 BeadChip	1	1036	-	1461
Di Gerlando et al. (13)	GoatSNP50 BeadChip	4	120	702	75
Yuan et al. (110)	Resequencing	3	32	1747604	7228
Salehian-Dehkordi et al. (66)	Ovine SNP600K BeadChip	67	2059	18152	1217
He et al. (64)	Resequencing	2	72	5862	-
Nandolo et al. (65)	Resequencing	34	82	253553	6231
Igoshin et al. (111)	Ovine Infinium HD SNP BeadChip	16	354	4527	1450
Taghizadeh et al. (68)	OvineSNP50 Beadchip	3	192	815	515
Salehian-Dehkordi et al. (112)	Ovine SNP600K BeadChip	47	695	39145	4769
Shi et al. (32)	Resequencing	2	20	60429	4927

### 3.3 Horses genome

China's horse industry has experienced remarkable growth owing to the country's rapid social and economic development, coupled with continuous improvements in living standards. Consequently, equestrian sports have gained significant popularity as leisure and recreational activities within the nation. The maturation of microarray and sequencing technologies has played a pivotal role in the identification of equine CNVs. These CNVs are of paramount importance for the study of equine trait variations, disease prevention and treatment, exploration of genetic diversity, tracing the origins of domestication, and the development of new equine breeds.

In recent years, the substantial progress in microarray and sequencing technologies has facilitated the detection of CNVs. Researchers from both domestic and international institutions have undertaken extensive investigations into equine genome CNV identification, with select research findings summarized in Table 3. Doan et al. (53) were the pioneers in reporting CNVs within equine genomes. They identified 775 CNV regions in 16 horses using an aCGH microarray, demonstrating the significant impact of CNVs on biological phenotypic diversity. Subsequently, in an effort to detect CNVs in both normal horses and Przewalski horses, Ghosh et al. (55) prepared an aCGH microarray that also included a Y chromosome probe. They successfully detected 258 CNVRs in autosomes, chrX, and chrUn, but none were found in chrY. Notably, the majority of these CNVRs were associated with genes related to sensory perception, the immune system, and reproduction (55). Previous study meticulously described the mapping of CNVs in Chinese horses using high-resolution array Comparative Genomic Hybridization (aCGH), a highly effective method for genome-wide CNV detection in animals (114). Consequently, Kader et al. (57) through whole-genome analysis of CNVs in 96 horses representing three Chinese breeds: Debo Shorthorn, Mongolian horse, and Yili horse. Their work identified a total of 287 CNVs, which were combined to form 122 CNV regions (CNVRs) with sizes ranging from 199 base pairs to 2,344 kilobases. Consistently, a study reported 15,041 CNVs and 5,350 CNVRs in 222 Friesian horses, creating a distribution map of CNVRs within the equine genome (115). Similarly, a recent study performed an analysis of CNV in 469 horses from four Korean breeds, uncovering 843 CNVRs that overlapped with 7.2% of the reference genome for horses (33). Furthermore, they constructed an autosomal map of CNVRs in horses. In addition, Laseca et al. (34) analyzed high-density SNP genotyping data from 654 horses, identifying a total of 19,902 CNV segments and 1,007 CNV regions, with CNVs covering 4.4% of the equine genome.

**Table 3:** Summary of CNVs and identification methods performed in horses.

**References**	**Methods**	**Species**	**Samples**	**CNVs**	**CNVRs**
Doan et al. (53)	aCGH arrays	15	16	2368	775
Dupuis et al. (113)	Equine SNP50 bead-chip array	4	477	2797	478
Metzger et al. (92)	Equine SNP50 bead-chip array	17	717	50	-
Ghosh et al. (55)	aCGH arrays	16	38	-	258
Wang et al. (114)	aCGH arrays	6	6	700	353
Kader et al. (57)	Equine SNP70 bead-chip array	3	96	287	122
Schurink et al. (115)	Equine genotyping array	1	222	15041	5350
Solé et al. (116)	Equine genotyping array	8	1755	18800	939
Al Abri et al. (61)	Resequencing	6	6	1540	-
Kim et al. (33)	Equine SNP70 bead-chip array	4	469	-	843
Laseca et al. (34)	Equine high-density 670K	1	654	19902	1007
Wang et al. (35)	Equine SNP70 bead-chip array	10	70	577	239
Choudhury et al. (37)	Resequencing	3	26	18974	4279

A study utilized gene chips to identify CNVs in 1,755 horses representing eight breeds. Their findings revealed that the size of CNV regions varied from 1 kilobase to 21.3 megabases (116). Consequently a study revealed an average total of 1,540 CNVs per horse through whole-genome resequencing of six horses representing six different breeds. Their results suggested that a reduction in the number of LATH copies might be linked to the development of endurance in horses (61). While a recent study by Choudhury et al. (37) leveraged resequencing data for Debao (DB), Baise (BS), and Warmblood (WB) horses to identify CNVs and create a CNVR map of the equine genome. Their research indicated that differential CNVRs may influence the phenotypic characteristics of different breeds.

### 3.4 Donkey genome

The donkey industry has emerged as a significant contributor to the growth of the livestock sector in recent years. However, the detection of CNVs within the genetic variations of the donkey genome, both in China and internationally, remains a relatively understudied area. In a study conducted by Han, a cohort comprising 263 native Chinese donkeys representing 13 breeds from eight provinces and regions was employed to identify CNVs in five Y chromosome genes of donkeys (CUL4BY, ETSTY1, ETSTY4, ETSTY5, and SRY) through quantitative Polymerase Chain Reaction (qPCR) analysis (58). While studies related to Single Nucleotide Polymorphisms (SNPs) have been reported, studies focusing on CNVs in donkeys are limited. Consistently, another study detected ~7 million SNPs in 126 domestic donkeys and made a noteworthy discovery that black or chestnut coat color was attributed to a 1 base pair deletion downstream of the TBX3 gene (117). This deletion led to reduced gene expression and its inhibitory effect on pigmentation. In addition to SNPs, CNVs represent crucial genetic resources. They possess distinct advantages, including their ubiquitous presence in the genome, extensive coverage, and relative ease of detection compared to SNPs (50, 118). The continued advancement of sequencing technology promises to greatly facilitate the identification of CNVs within the donkey genome.

The subsequent section offers insights into potential reasons why CNVs have been relatively overlooked in donkey research:

1. Donkeys predominantly serve as working animals in many parts of the world. Consequently, researchers may prioritize the study of genetic markers associated with phenotype traits in species other than donkeys.
2. Initially, copy number variation was primarily detected using gene chips, which were less readily available for donkeys compared to other livestock species such as cattle, sheep, and horses. In recent years, the development of sequencing technology and the completion of whole-genome sequencing for donkeys have created favorable conditions for the detection of CNVs within the donkey genome.
3. Funding for research pertaining to donkeys may be comparatively constrained when compared to other livestock species like cattle and sheep. Consequently, researchers may face challenges in securing adequate resources for the study of CNVs in donkeys.
4. In many parts of the world, the commercial value of donkeys is primarily linked to their performance as working animals, rather than their utility for products such as milk or meat. As a result, there may be limited commercial interest in investigating CNVs in donkeys.

## 4 Gene ontology analysis for genes overlapping CNVRs

To identify genes that may be influenced by CNVRs within the genome, an annotation analysis of genes associated with CNVRs revealed that olfactory-related functions, specifically olfactory transduction and olfactory receptor activity, were frequently affected. This observation aligns with previous findings in various species, including cattle (119), sheep (62, 111), horses (34, 115, 120) and humans (121–123), where genes affected by copy number variation have shown enrichment for olfactory-related functions. In the case of humans, the sense of smell is considered a minor aspect of overall health and may not be closely linked with adaptation. Consequently, human olfactory receptor (OR) genes tend to evolve neutrally (121). However, in the animal kingdom, the sense of smell holds paramount importance as it plays a vital role in locating food, identifying harmful substances, avoiding predators, selecting mates, and ensuring long and healthy survival and reproduction (124). Consistently, a study proposed that olfactory receptors also play a role in appetite regulation and feeding efficiency in mammals. Hence, alterations in these receptors may lead to individual differences in feed intake, body weight, and body composition (125). Additionally, CNVRs have been found to intersect with Quantitative Trait Loci (QTL) associated with various factors such as morphology, disease resistance, and more. This intersection serves as a fundamental foundation for the examination of phenotypic diversity. Variations in CNVR frequency among different breeds have been identified in CNVR tests involving animals from various regions (52, 111, 126). These variations can be attributed to breed domestication and environmental adaptation.

## 5 CNVs and their association with phenotypic traits in herbivorous livestock

It has been well-established that genomic CNVs exert an impact on an organism's phenotype through various mechanisms, including changes in gene dosage, modulation of gene expression, modulation of gene transcriptional regulators, and positional effects (127). The association of CNVs in genes and their association with various phenotypic traits (growth traits, reproductive traits, pigmentation, and diseases resistance) in herbivorous livestock (cattle, sheep, goat, and horses) have been summarized in Table 4.

**Table 4:** Summary of CNVs in genes and their association with phenotypic traits in herbivorous livestock.

**Genes (CNVs)**	**Phenotypes**	**Biological effect**	**Species**	**References**
Changes in the CNV region within the LEPR intron 3	Growth traits	Body weight, body height, body length, and brisket circumference	Cattle	(128)
Myosin heavy chain 3 (MYH3)-CNV		Skeletal muscle development		(129)
Mitogen-activated protein kinase 10 (MAPK10)-CNV		Body weight (*P < * 0.05), body height and chest girth		(130)
CYP4A11-CNV		Lipid deposition		(131)
Guanylate binding protein 2 (GBP2)-CNV		Body height, body length, heart girth, hip width, rump length		(132)
Insulin-like growth factor 1 receptor (IGF1R)-CNV		Associated with body weight and body height of Jinnan cattle and was significantly linked with body height and hucklebone width of Qinchuan cattle		(133)
Kupple like factor 3 (*KLF3*)-CNV		Body mass and heart girth		(134)
Potassium inwardly-rectifying channel, subfamily J 12 (KCNJ12)-CNV (1&2)		Significant association with the body length, chest circumference, body weight, rump length		(135)
MLLT10-CNV		Hip width, rump length, hucklebone width, and cannon bone circumference		(136)
Uanylate-binding protein 6 (GBP6)-CNV		Associated with body weight, cannon circumference and chest circumference		(137)
(CNV1: 3600 bp, including exon 2–11; CNV2: 4800 bp, including exon 21–22) of the CLCN2 gene		Cannon circumference, body slanting length, chest girth, and body weight		(138)
CCDC39- CNV (Normal, deletion, duplication)		body length, hip width, heart girth and cannon bone, and circumference		(139)
PLA2G2A-CNV *(Normal, Deletion)*		Height at sacrum, heart girth and body height, chest depth		(140)
SYT11-CNV		Significantly correlated with body length, cannon circumference, chest depth, rump length, and forehead size of Yunling cattle, and was significantly correlated with the bodyweight of Xianan cattle		(141)
Mitochondrial fusion protein (MFN1)-CNV		Significant correlation with hucklebone width, hip width, height at sacrum, chest width and rump length		(142)
DYNC1I2-CNV (Duplication and deletion)		Associated with height at hip cross, body length, chest width and hucklebone width, chest depth		(143)
WW domain binding protein 1-like (WBP1L)- CNV		Associated with heart girth, rump length and body weight (Pinan cattle), withers height, rump length, body length, chest depth and BW of (Jiaxian cattle)		(144)
MUC19-CNV		Correlated with hip width, height at hip cross and withers height, body length, and huckle bone width		(145)
SERPINA3-1-CNV		Body height		(12)
GAL3ST1- CNV (deletion)		Body weight		(146)
VAMP7-Duplication		Body growth trait (height at the hip cross)		(147)
ZNF679-CNV		Body size and length		(148)
CNVRs harbored genes (PPARA, RXRA, KLF11, ADD1, FASN, PPP1CA, PDGFA, and PEX6)		Fat deposition	Sheep	(107)
Src homology 2 domain containing E (SHE)-CNV		Correlated to body length, circumference of cannon bone, heart girth, chest width and high at the cross		(149)
ORMDL sphingolipid biosynthesis regulator 1 (ORMDL1)-CNV		Body weight, body height, body length, chest depth, and height of hip cross		(150)
KMT2D-CNV		Body length, withers height, hip width		(151)
TOP2B-CNV		Body length, chest circumference, canon circumference and height of hip cross		(152)
BAG4 -CNV		Body height, body slanting length, body height and hip cross height		(153)
TOP2B-CNV		Body length, chest circumference, canon circumference and height of hip cross		(152)
LRRFIP1-CNV		Chest width, rump breadth and circumference of cannon, larger heart girth		(154)
KAT6A-(CNV1, CNV2, CNV3)		Body height and body length		(155)
PIGY- CNV		Body weight, chest circumference, and tube circumference		(156)
Myosin light chain kinase-4(MYLK)-CNV		Body weight, body length and body height	Goat	(157)
Opn4-CNV		Body weight in Guizhou white goat Body length in Guizhou black goat		(158)
SNX29 gene-CNV ADCY1-CNV		Meat production traits		(159)
CADM2-CNV (Deletion)		Withers height and body length		(160)
Sorting nexin 29 (SNX29)-CNV		Body length, body height, heart girth, chest width, canon circumference		(161)
Myogenic differentiation 1 (MyoD1)-CNV		Body weight, height at hip cross, heart girth and hip width		(30)
A-kinase-anchoring protein 13 (AKAP13)-CNV		Body height and body length, chest depth, chest circumference, and cannon circumference		(162)
Pleomorphic adenoma gene 1 (PLAG1)-CNV		Body weight, heart girth, height at hip cross, and hip width		(163)
CCSER1-CNV (deletion)		Body weight and heart girth traits		(29)
Deleted regions on ECA1, ECA8 and ECA9.		Body height	Horses	(92)
23 CNVRs with overlapping QTLs associated with equine body height		Body height		(33)
A deletion in the intronic region of the SPAG16 gene	Reproductive traits	Bull-fertility traits (sperm motility)	Cattle	(104)
CNV of ZNF280BY		Negative correlation with the percentage of normal sperm and sperm concentration		(164)
CNVs of the bovine HSFY and ZNF280BY		Correlated negatively with testis size, while positively with sire conception rate.		(165)
CNV of *ZNF280BY*		Negatively correlated with testis size	Hu sheep	(166)
SMAD2		Litter size and semen quality	Goat	(40)
Sorting nexin 29 (SNX29)-CNV (Indel)		Litter size and fertility		(167)
Protein phosphatase 3 catalytic subunit alpha (PPP3CA)-CNV		Litter size and semen quality		(168)
PRP1 and PRP6 have CNV mutations in the HF group		Litter size		(169)
CNVs in regions of the Y chromosome		Male development and equine fertility	Horses	(170)
An 1155 bp deletion in the ASIP gene		Coat color darkening		(171)
2,809 bp LINE-1 insertion in ASIP gene		White coat color		(172)
ASIP-CNV (Duplication)		Light coat color	Goat	(56)
ASIP- CNV		White coat colounr		(173)
1 Mb CNV affect EDNRA gene		White coat color		(174)
13.42kb duplication upstream of ASIP		Non-classic swiss markings		(175)
A 4.6 kb duplication in intron 6 of STX17		Gray phenotype	Horses	(176)
A deletion including exon 3 of the ED1 gene	Disease resistance	Anhidrotic Ectodermal Dysplasia	Cattle	(177)
Low relative expression levels of KIF2A and PHKG2		Disease resistance	Sheep	(106)
CNVS in CCL1, CCL2, CCL8, CCL11, NOS2, TNF, CSF3, and STAT3 genes		Resistance to natural *Haemonchus contortus* infections		(41)
CNVR33, CNVR65, and CNVR7 overlap with immune system-related genes		Strong resistance to infectious diseases	Goat	(13)
CNVRs located in the MHC region of ECA20		Insect bite hypersensitivity	Horses	(115)
A pure deletion of the AKR1C gene		Disorders of sexual development		(55)
CNV in GRIK4, IFNLR1, and LOC102275985	Environmental adaptability	High-altitude adaptation	Cattle	(178)
CNV in LDHB and ME1		Cold and low oxygen environments		(179)
CNV changes affect in ALKBH5, NARFL genes		Plateau acclimatization	Sheep	(107)
CNVR is significantly correlated with solar radiation		Solar radiation		(112)
Genes associated with hemoglobin binding located on CNVRs		Harsh plateau environment	Horses	(114)
Changes in NFKBIA, SOCS4, HSPA1A, and IL6 genes located in the CNVR		High temperatures and humidity		(35)

### 5.1 CNVs associated with growth and reproductive traits in herbivorous livestock

CNVs have been extensively studied in various livestock species, including cattle, sheep, donkey and horses, and its association with important growth, reproduction, and fertility traits has been documented. This comprehensive review discusses key findings and contributions from various research studies in these livestock species. In cattle, Yang et al. (131) identified CNV in the cytochrome P-450 4A11 (CYP4A11) gene, which was associated with increased growth. Multiple copies of CYP4A11 were found to promote the differentiation of 3T3-L1 cells into adipocytes, potentially leading to increased fat deposition. Previous studies investigated SERPINA3-1 and GAL3ST1 gene CNVs in different Chinese cattle breeds, revealed associations with growth traits such as body height, body weight, and rump width (146, 180). These genes hold promise as candidate genes for Chinese cattle breeding. Correspondingly, Hu et al. (139) analyzed CCDC39 gene CNVs and their impact on body length and hip width, noting significant effects, particularly in the Pinan (PN) breed. Additionally, Shi et al. (128) found a correlation between leptin gene CNV and various phenotypic traits, including body weight, body height, body length, and brisket circumference, in multiple cattle breeds.

In sheep, Zhu et al. (107) identified adiposity-related genes, including PPARA, RXRA, KLF11, ADD1, FASN, PPP1CA, and PDGFA, in CNV regions of fat-tailed sheep. These genes were associated with fat deposition, with individuals carrying copy number deletions exhibiting higher body weight. Similarly, a recent study by Wang et al. (155) documented a significant correlation between CNV in the KAT6A gene and body height and hip width in Hu sheep (HU). They also found that CNV3 duplicates were associated with higher body height and weight. Yang et al. (153) highlighted the BAG4 gene's role in regulating body height in sheep and its potential as a molecular marker for molecular breeding. Feng et al. (156) discovered that CNVs of the PIGY gene significantly impacted body weight, chest circumference, and tube circumference in sheep. Additionally, Xu et al. (29) found that CNV types of the CCSER1 gene were correlated with body weight and heart girth traits in Guizhou white goats (GZW).

Horses were also subject to CNV studies. Consistently, Metzger et al. (92) conducted a genome-wide analysis of CNVs and their association with equine body height traits. They identified deleted regions in ECA1, ECA8, and ECA9, which were significantly linked to equine body height. While Kim et al. (33) reported CNVRs with overlapping quantitative trait loci (QTLs) associated with equine body height in Jeju riding horses and Hanra horses. These findings provided valuable insights into the genetic factors influencing equine body height.

In the context of fertility, CNVs have also been explored. Consistently, a deletion in the intronic region of the SPAG16 gene has been identified in bulls with poor sperm motility (PSM), suggesting its potential role in bull fertility (104). A comprehensive analysis documented CNVs in Laoshan dairy goat populations with differing fertility levels and showed that CNV mutations in PRP1 and PRP6 genes, which affect mammalian fertility (169). A study investigated CNVs in Y chromosome-specific regions in male horses, identifying potential genes linked to stallion fertility and contributing to our understanding of equine male development and fertility (170). These studies collectively underscore the significance of CNVs in shaping various traits in cattle, sheep, and horses, from growth and fat deposition to fertility. The identification of specific genes and regions associated with these traits holds promise for selective breeding programs and further genetic research in these livestock species.

### 5.2 Pigmentation

The role of CNVs in determining coat color and disease resistance in cattle, horses, and sheep has been the subject of extensive scientific investigation. In this discussion, we will delve into various studies that have shed light on the influence of CNVs on these traits in these livestock species. A study reported two sequence translocations between chromosomes 6 and 29 in Belgian Blue and Swiss Brown cattle, affecting the KIT gene, leading to color-sidedness (181). Similarly another study reported that a deletion of 1155 bp within the ASIP gene in Nellore cattle results in dark hair color in specific regions by elevating melanin production (171). Furthermore, a 2,809 bp LINE-1 insertion in the ASIP gene icausing a white coat color phenotype by impeding melanin production has been identified in buffalo (172). Consequently a study postulated that a 4.6 kb duplication within intron 6 of the STX17 gene leads to a pronounced upregulation of both STX17 and NR4A3 gene expression (176). This heightened gene expression subsequently instigates the proliferation of melanocytes, ultimately culminating in the manifestation of a gray coat phenotype in affected horses. Furthermore, it is noteworthy that horses harboring this mutant phenotype exhibit a gradual transition in hair color from gray to white as they advance in age. In a similar vein, previous investigations encompassed genomic analyses of feral and domestic goat populations, which unveiled the intriguing revelation that 13 genes situated within CNV regions overlap with the comprehensive roster of cloned color genes provided by the European Society for Pigment Cell Research (ESPCR) (56). Moreover, these investigations substantiated the substantial impact of CNVs within the ASIP gene on the lightening of coat color in domestic goat breeds, employing rigorous resequencing analyses. A subsequent inquiry brought to light the potential influence of CNVs on the ASIP gene, which may, in turn, lead to the emergence of a white coat color phenotype in the Girgentana and Saanen goat breeds, as corroborated by Fontanesi et al. (173). A recent report presented compelling evidence demonstrating the significant association between a 13.42 kb repeat sequence located upstream of the ASIP gene and non-classic Swiss markings in goats, utilizing CNV assays and quantitative Polymerase Chain Reaction (qPCR) techniques (175). This finding further underscores the pivotal role of CNVs in shaping coat color patterns in goats. Furthermore, Menzi et al. (174) unraveled the involvement of the EDNRA gene, situated within a 1 Mb CNV region on chromosome 17, in potentially attenuating melanism among Boer goats. Notably, an elevation in the copy number within this CNV region could potentially lead to a reduction in skin pigmentation, thereby culminating in the manifestation of a white-spotted phenotype. In summary, these comprehensive investigations collectively shed light on the intricate interplay between CNVs and the genetic determinants of coat color diversity in horses and goats, providing valuable insights into the underlying genetic mechanisms governing these phenotypic variations.

### 5.3 Disease resistance

The CNVs hold significant potential to influence disease resistance in livestock species, including cattle, sheep, and horses. Notably, Liu et al. (52) conducted an exhaustive investigation in cattle, uncovering multiple CNVs that play pivotal roles in crucial biological processes such as drug detoxification, innate and adaptive immunity, as well as receptor and signal recognition. In the context of cattle, it is worth highlighting the association between CNVs and disease susceptibility. For instance, Drögemüller et al. (177) reported that a deletion encompassing exon 3 of the ED1 gene has been linked to anhidrotic ectodermal dysplasia, underscoring the critical role of CNVs in disease vulnerability. Furthermore, a study reported CNVs in 18 candidate genes (TERT, NOTCH1, SLC6A3, CLPTM1L, PPARα, BCL-2, ABO, VAV2, CACNA1S, TRAF2, RELA, ELF3, DBH, CDK5, NF2, FASN, EWSR1 and MAP3K11) which were associated with milk somatic cells count and mastitis resistance in dairy cattle (182). Consistently, another study reported that CNVs in ZNF496 and NLRP3 were significantly associated with resistance to gastrointestinal nematodes in Angus cattle (129).

Shifting our focus to small ruminants, Di Gerlando et al. (13) embarked on an exploration of Sicilian goat breeds. Their study unveiled intriguing findings, as CNVR33, CNVR65, and CNVR7 were found to overlap with genes closely associated with the immune system. This discovery offers a potential explanation for the remarkable resistance of these goat breeds against infectious diseases. In the case of Florida Native sheep, Estrada-Reyes et al. (41) delved into the genetic underpinnings of resistance against gastrointestinal nematodes. Their meticulous investigation revealed that 14 CNVs exhibited overlaps with QTLs associated with gastrointestinal nematode resistance. Moreover, these CNVs demonstrated significant correlations with fecal egg count (FEC), suggesting a potential influence of CNVs on parasite resistance in these sheep. Ma et al. (106) conducted an extensive study encompassing 160 Chinese sheep breeds, leading to the identification of 111 CNV regions. Their functional analysis highlighted an enrichment of CNV regions with genes closely linked to environmental responses. Notably, 17 candidates genes emerged from this analysis, primarily associated with specific diseases, metabolic processes, and development. Particularly intriguing was the observation of lower relative expression levels of KIF2A and PHKG2 in domestic sheep breeds compared to introduce sheep breeds, implying enhanced disease resistance within modern Gansu sheep breeding populations.

In the equine domain, Schurink et al. (115) undertook a thorough investigation into CNVs within Friesian horses. Their research unveiled a staggering 15,041 CNVs identified across 222 individuals. Importantly, this study integrated genome-wide association study (GWAS) leveraging both SNPs and CNVs data. A significant finding was the association between CNV regions situated in the major histocompatibility complex (MHC) region of ECA20 and insect bite hypersensitivity (IBH) in Friesian horses. Notably, approximately half of the horses included in the study were afflicted by this condition. Consistently, Ghosh et al. (55) conducted a comprehensive genomic analysis encompassing healthy horses representing 16 distinct breeds. Their investigation identified 258 CNV regions. Notably, the study extended its inquiry to horses exhibiting sexual developmental impairments, wherein they identified a pure deletion of the AKR1C gene in two male pseudohermaphrodites. This discovery suggests a potential association between this gene deletion and the observed abnormalities.

### 5.4 Environmental adaptability

A comparative examination of CNVs in herbivorous livestock originating from diverse regional breeds has illuminated the potential influence of CNVs on their environmental adaptability. A performed a comprehensive investigation into the differential distribution of CNVs within yak populations hailing from the Tibet and Gansu regions (178). Their study identified seven candidate CNVs, specifically annotating three genes (GRIK4, IFNLR1, and LOC102275985) enriched in five well-established signaling pathways that play pivotal roles in the animals' acclimatization to their environments and various physiological functions. Of particular note is the regulation of physiological processes in hypoxic environments. This research significantly contributes to our understanding of the molecular mechanisms underlying the high-altitude environmental adaptability of yaks. Qaidam cattle, known for their adaptation to cold and low-oxygen environments, underwent genomic analysis by Guo et al. (179). This study identified LDHB and ME1 as potential key genes influencing the cattle's remarkable adaptability to such harsh conditions.

Tibetan sheep, native to high-altitude plateaus, were investigated by Zhu et al. (107), who identified 66 CNVRs associated with their plateau acclimatization. Notably, α-ketoglutarate-dependent dioxygenase alkB homolog 5 (ALKBH5) and nuclear prelamin A recognition factor-like (NARFL) were found to be associated with plateau adaptation within the identified CNVRs. A study performed by Salehian-Dehkordi et al. (112) documented CNVs in 47 sheep breeds. Their research revealed 155 CNVs highly significantly correlated with various environmental parameters, with 35 CNVRs showing significant correlations with solar radiation. Moreover, genes overlapping with CNVs, such as B3GNTL1, UBE2L3, TRAF2, GTF2F1, and IGFALS, were significantly correlated with climatic variables, further emphasizing the role of CNVs in environmental adaptation.

In the equine domain, Wang et al. (114) utilized aCGH to identify CNVs in six horse breeds, uncovering a total of 700 CNVs. These CNVs were classified into 353 CNVRs, and their genetic examination revealed specific genes associated with hemoglobin binding, suggesting a potential influence on horses' adaptability to the challenging plateau environment. The Jinjiang horse, an indigenous breed exclusive to the southeastern coast of China and adapted to high temperatures and humidity, was the focus of a study by Wang et al. (35). They identified 229 genes that overlapped with CNVRs, with four specific candidate genes (NFKBIA, SOCS4, HSPA1A, and IL6) highlighted due to their strong correlation with cellular thermal acclimatization.

## 6 Conclusions

Common herbivorous livestock, like cattle and sheep, are vital to human daily life due to their significant contributions to meat and dairy production, playing crucial roles in animal husbandry. Recently, China has seen remarkable growth in its equine and donkey industries, driven by advancements in science, technology, and societal progress, establishing themselves as emerging specialties within the livestock sector. Therefore, investigating CNVs within the genomes of these animals holds profound importance. Advancements in microarray and sequencing technologies, along with decreasing sequencing costs, have provided a robust foundation for identifying and studying CNVs. These CNVs, characterized by extended mutant fragment lengths and their substantial impact on genes, represent a formidable genetic resource for exploring genetic variations in livestock and poultry. Extensive research focusing on CNVs in herbivore genomes has unequivocally demonstrated the pivotal role CNVs play in shaping phenotypic diversity, influencing economic traits, enhancing disease resistance, and facilitating environmental adaptation. Researchers worldwide have dedicated their efforts to elucidating the connection between CNVs and phenotypic differences as well as diseases in livestock and poultry. These findings offer compelling support for exploring the potential applications of CNVs as genetic markers in regulation of various productive and disease resistance traits. Consequently, CNVs emerge as a promising avenue for augmenting genetic diversity and expediting molecular breeding strategies in common herbivorous livestock, including cattle, sheep, horses, and donkeys. In conclusion, CNVs represent a valuable and dynamic field of study poised to make a lasting impact on the genetic improvement of herbivorous livestock species, ultimately benefiting both human society and the global livestock industry.

## Author contributions

XL: Conceptualization, Data curation, Formal analysis, Funding acquisition, Investigation, Methodology, Software, Writing—original draft, Writing—review & editing. WChe: Methodology, Writing—review & editing, Data curation, Formal analysis. BH: Writing—review & editing, Investigation, Software. XW: Investigation, Software, Writing—review & editing, Data curation, Formal analysis. YP: Data curation, Software, Writing—review & editing, Conceptualization. XZ: Data curation, Software, Writing—review & editing, Investigation. WCha: Software, Writing—review & editing, Formal analysis, Methodology. MK: Methodology, Writing—review & editing, Conceptualization, Project administration, Supervision, Writing—original draft. CW: Conceptualization, Funding acquisition, Investigation, Methodology, Project administration, Resources, Supervision, Validation, Visualization, Writing—original draft, Writing—review & editing.

## References

[1] Hancock JF. Origins of World Crops and Livestock, World Agriculture Before and After 1492: Legacy of the Columbian Exchange. Cham: Springer (2022), 5–18.

[2] Caldwell CD. Domestication in agricultural systems In CD Caldwell, S Wang, editors. Introduction to Agroecology. Singapore: Springer Singapore, 157–67.

[3] Andersson L Purugganan M. Molecular genetic variation of animals and plants under domestication. Proc Nat Acad Sci. (2022) 119:e2122150119. 10.1073/pnas.2122150119 35858409 PMC9335317

[4] Verbiest M Maksimov M Jin Y Anisimova M Gymrek M Bilgin Sonay T. Mutation and selection processes regulating short tandem repeats give rise to genetic and phenotypic diversity across species. J Evol Biol. (2023) 36:321–36. 10.1111/jeb.14106 36289560 PMC9990875

[5] Lee YL Bosse M Takeda H Moreira GC Karim L Druet T. High-resolution structural variants catalogue in a large-scale whole genome sequenced bovine family cohort data. BMC Genomics. (2023) 24:1–7. 10.1186/s12864-023-09259-8 37127590 PMC10152703

[6] Khan MZ Wang J Ma Y Chen T Ma M Ullah Q. Genetic polymorphisms in immune-and inflammation-associated genes and their association with bovine mastitis resistance/susceptibility. Front Immunol. (2023) 14:1082144. 10.3389/fimmu.2023.1082144 36911690 PMC9997099

[7] Khan IM Khan A Liu H Khan MZ. Genetic markers identification for animal production and disease resistance. Front Genet. (2023) 14:1243793. 10.3389/fgene.2023.1243793 37501722 PMC10369344

[8] Wang T Wang X Liu Z Shi X Ren W Huang B. Genotypes and haplotype combination of DCAF7 gene sequence variants are associated with number of thoracolumbar vertebrae and carcass traits in Dezhou donkey. J Appl Anim Res. (2023) 51:31–9. 10.1080/09712119.2022.2149538

[9] Khan MZ Ma Y Ma J Xiao J Liu Y Liu S. Association of DGAT1 with cattle, buffalo, goat, and sheep milk and meat production traits. Frontiers in Veterinary Science. (2021) 8:712470. 10.3389/fvets.2021.712470 34485439 PMC8415568

[10] Weischenfeldt J Symmons O Spitz F Korbel JO. Phenotypic impact of genomic structural variation: insights from and for human disease. Nat Rev Genet. (2013) 14:125–38. 10.1038/nrg3373 23329113

[11] Begna T. Role and economic importance of crop genetic diversity in food security. Int J Agric Sci Food Technol. (2021) 7:164–9. 10.17352/2455-815X.000104

[12] Huang B Khan MZ Chai W Ullah Q Wang C. Exploring genetic markers: mitochondrial dna and genomic screening for biodiversity and production traits in donkeys. Animals. (2023) 13:2725. 10.3390/ani13172725 37684989 PMC10486882

[13] Di Gerlando R Mastrangelo S Moscarelli A Tolone M Sutera AM Portolano B. Genomic structural diversity in local goats: analysis of copy-number variations. Animals. (2020) 10:1040. 10.3390/ani10061040 32560248 PMC7341319

[14] Haque MA Alam MZ Iqbal A Lee YM Dang CG Kim JJ. Genome-wide association studies for body conformation traits in korean holstein population. Animals. (2023) 13:2964. 10.3390/ani13182964 37760364 PMC10526087

[15] Wang P Li X Zhu Y Wei J Zhang C Kong Q. Genome-wide association analysis of milk production, somatic cell score, and body conformation traits in Holstein cows. Front Vet Sci. (2022) 9:932034. 10.3389/fvets.2022.932034 36268046 PMC9578681

[16] Yang P Wang G Jiang S Chen M Zeng J Pang Q. Comparative analysis of genome-wide copy number variations between Tibetan sheep and White Suffolk sheep. Anim Biotechnol. (2023) 34:986–93. 10.1080/10495398.2021.2007937 34865600

[17] Sölzer N May K Yin T König S. Genomic analyses of claw disorders in Holstein cows: genetic parameters, trait associations, and genome-wide associations considering interactions of SNP and heat stress. J Dairy Sci. (2022) 105:8218–36. 10.3168/jds.2022-22087 36028345

[18] Junior LP Pinto LF Cruz VA Junior GA Oliveira HR Chud TS. Genome-wide association and functional genomic analyses for various hoof health traits in North American Holstein cattle. J Dairy Sci. (2023). 37939841 10.3168/jds.2023-23806

[19] Schneider H Segelke D Tetens J Thaller G Bennewitz J. A genomic assessment of the correlation between milk production traits and claw and udder health traits in Holstein dairy cattle. J Dairy Sci. (2023) 106:1190–205. 10.3168/jds.2022-22312 36460501

[20] Hay EH Utsunomiya YT Xu L Zhou Y Neves HH Carvalheiro R. Genomic predictions combining SNP markers and copy number variations in Nellore cattle. BMC Genomics. (2018) 19:1–8. 10.1186/s12864-018-4787-6 29871610 PMC5989480

[21] Sudmant PH Rausch T Gardner EJ Handsaker RE Abyzov A Huddleston J. An integrated map of structural variation in 2,504 human genomes. Nature. (2015) 526:75–81. 10.1038/nature15394 26432246 PMC4617611

[22] Fadista J Thomsen B Holm L Bendixen C. Copy number variation in the bovine genome. BMC Genomics. (2010) 11:284. 10.1186/1471-2164-11-284 20459598 PMC2902221

[23] Stothard P Choi J Basu U Sumner-Thomson JM Meng Y Liao X. Whole genome resequencing of black angus and holstein cattle for snp and cnv discovery. BMC Genomics. (2011) 12:559. 10.1186/1471-2164-12-559 22085807 PMC3229636

[24] Butty AM Chud TCS Cardoso DF Lopes LSF Miglior F Schenkel FS. Genome-wide association study between copy number variants and hoof health traits in holstein dairy cattle. J Dairy Sci. (2021) 104:8050–61. 10.3168/jds.2020-19879 33896633

[25] Alkan C Coe BP Eichler EE. Genome structural variation discovery and genotyping. Nat Rev Genet. (2011) 12:363–76. 10.1038/nrg2958 21358748 PMC4108431

[26] Butty AM Chud TCS Miglior F Schenkel FS Kommadath A Krivushin K. High confidence copy number variants identified in holstein dairy cattle from whole genome sequence and genotype array data. Sci Rep. (2020) 10:8044. 10.1038/s41598-020-64680-3 32415111 PMC7229195

[27] Braga LG Chud TC Watanabe RN Savegnago RP Sena TM. Identification of copy number variations in the genome of Dairy Gir cattle. PLoS ONE. (2023) 18:e0284085. 10.1371/journal.pone.0284085 37036840 PMC10085049

[28] Liu Y Mu Y Wang W Ahmed Z Wei X Lei C. Analysis of genomic copy number variations through whole-genome scan in Chinese Qaidam cattle. Front Vet Sci. (2023) 10:1148070. 10.3389/fvets.2023.1148070 37065216 PMC10103646

[29] Xu Z Wang X Song X An Q Wang D Zhang Z. Association between the copy number variation of ccser1 gene and growth traits in chinese capra hircus (goat) populations. Anim Biotechnol. (2023) 34:1377–83. 10.1080/10495398.2022.2025818 35108172

[30] Ren H Wei Z Li X Wang Q Chen H Lan X. Goat MyoD1: mRNA expression, InDel and CNV detection and their associations with growth traits. Gene. (2023) 866:147348. 10.1016/j.gene.2023.147348 36898510

[31] Yao Z Zhang S Wang X Guo Y Xin X Zhang Z. Genetic diversity and signatures of selection in BoHuai goat revealed by whole-genome sequencing. BMC Genomics. (2023) 24:116. 10.1186/s12864-023-09204-9 36922782 PMC10018941

[32] Shi H Li T Su M Wang H Li Q Lang X. Identification of copy number variation in tibetan sheep using whole genome resequencing reveals evidence of genomic selection. BMC Genomics. (2023) 24:555. 10.1186/s12864-023-09672-z 37726692 PMC10510117

[33] Kim Y Ha S Seong H Choi J Baek H Yang B. Identification of copy number variations in four horse breed populations in south korea. Animals. (2022) 12. 10.3390/ani12243501 36552421 PMC9774267

[34] Laseca N Molina A Valera M Antonini A Demyda-Peyrás S. Copy number variation (cnv): a new genomic insight in horses. Animals. (2022) 12:1435. 10.3390/ani12111435 35681904 PMC9179425

[35] Wang M Liu Y Bi X Ma H Zeng G Guo J. Genome-wide detection of copy number variants in chinese indigenous horse breeds and verification of cnv-overlapped genes related to heat adaptation of the jinjiang horse. Genes. (2022) 13:603. 10.3390/genes13040603 35456409 PMC9033042

[36] Zandi MB Salek Ardestani S Vahedi SM Mahboudi H Mahboudi F Meskoob A. Detection of common copy number of variants underlying selection pressure in middle eastern horse breeds using whole-genome sequence data. J Heredity. (2022) 113:421–30. 10.1093/jhered/esac027 35605262

[37] Choudhury MP Wang Z Zhu M Teng S Yan J Cao S. Genome-wide detection of copy number variations associated with miniature features in horses. Genes. (2023) 14:1934. 10.3390/genes14101934 37895283 PMC10606273

[38] Castaneda C Radović L Felkel S Juras R Davis BW Cothran EG Wallner B Raudsepp T. Copy number variation of horse Y chromosome genes in normal equine populations and in horses with abnormal sex development and subfertility: relationship of copy number variations with Y haplogroups. G3 J. (2022) 12:jkac278. 10.1093/g3journal/jkac278 36227030 PMC9713435

[39] Sun X Jiang J Wang G Zhou P Li J Chen C. Genome-wide association analysis of nine reproduction and morphological traits in three goat breeds from Southern China. Anim Biosci. (2023) 36:191. 10.5713/ab.21.0577 35760404 PMC9834730

[40] Wijayanti D Luo Y Bai Y Pan C Qu L Guo Z. New insight into copy number variations of goat SMAD2 gene and their associations with litter size and semen quality. Theriogenology. (2023) 206:114–22. 10.1016/j.theriogenology.2023.05.012 37229957

[41] Estrada-Reyes ZM Ogunade IM Pech-Cervantes AA Terrill TH. Copy number variant-based genome wide association study reveals immune-related genes associated with parasite resistance in a heritage sheep breed from the united states. Parasite Immunol. (2022) 44:e12943. 10.1111/pim.12943 36071651 PMC9786709

[42] Lovett PS Duvall EJ Keggins KM. Bacillus pumilus plasmid ppl10: properties and insertion into bacillus subtilis 168 by transformation. J Bacteriol. (1976) 127:817–28. 10.1128/jb.127.2.817-828.1976 821919 PMC232989

[43] Buckler CE Staal SP Rowe WP Martin MA. Variation in the number of copies and in the genomic organization of ecotropic murine leukemia virus proviral sequences in sublines of akr mice. J Virol. (1982) 43:629–40. 10.1128/jvi.43.2.629-640.1982 6287036 PMC256165

[44] Hollox EJ Armour JAL Barber JCK. Extensive normal copy number variation of a β-defensin antimicrobial-gene cluster. The Am J Hum Genetics. (2003) 73:591–600. 10.1086/378157 12916016 PMC1180683

[45] Pollack JR Sørlie T Perou CM Rees CA Jeffrey SS Lonning PE. Microarray analysis reveals a major direct role of dna copy number alteration in the transcriptional program of human breast tumors. Proc Nat Acad Sci. (2002) 99:12963–8. 10.1073/pnas.162471999 12297621 PMC130569

[46] Lucito R Healy J Alexander J Reiner A Esposito D Chi M. Representational oligonucleotide microarray analysis: a high-resolution method to detect genome copy number variation. Genome Res. (2003) 13:2291–305. 10.1101/gr.1349003 12975311 PMC403708

[47] Guillaud-Bataille M Valent A Soularue P Perot C Inda M Receveur A. Detecting single dna copy number variations in complex genomes using one nanogram of starting dna and bac-array cgh. Nucleic Acids Res. (2004) 32:e112. 10.1093/nar/gnh108 15284333 PMC506828

[48] Sebat J Lakshmi B Troge J Alexander J Young J Lundin P. Large-scale copy number polymorphism in the human genome. Science. (2004) 305:525–8. 10.1126/science.1098918 15273396

[49] Chen Q Book M Fang X Hoeft A Stuber F. Screening of copy number polymorphisms in human β-defensin genes using modified real-time quantitative pcr. J Immunol Methods. (2006) 308:231–40. 10.1016/j.jim.2005.11.001 16380128

[50] Redon R Ishikawa S Fitch KR Feuk L Perry GH Andrews TD. Global variation in copy number in the human genome. Nature. (2006) 444:444–54. 10.1038/nature05329 17122850 PMC2669898

[51] Matukumalli LK Lawley CT Schnabel RD Taylor JF Allan MF Heaton MP. Development and characterization of a high density snp genotyping assay for cattle. PLoS ONE. (2009) 4:e5350. 10.1371/journal.pone.0005350 19390634 PMC2669730

[52] Liu GE Hou Y Zhu B Cardone MF Jiang L Cellamare A. Analysis of copy number variations among diverse cattle breeds. Genome Res. (2010) 20:693–703. 10.1101/gr.105403.110 20212021 PMC2860171

[53] Doan R Cohen N Harrington J Veazy K Juras R Cothran G. Identification of copy number variants in horses. Genome Res. (2012) 22:899–907. 10.1101/gr.128991.111 22383489 PMC3337435

[54] Liu J Zhang L Xu L Ren H Lu J Zhang X. Analysis of copy number variations in the sheep genome using 50k snp beadchip array. BMC Genomics. (2013) 14:229. 10.1186/1471-2164-14-229 23565757 PMC3626776

[55] Ghosh S Qu Z Das PJ Fang E Juras R Cothran EG. Copy number variation in the horse genome. PLoS Genet. (2014) 10:e1004712. 10.1371/journal.pgen.1004712 25340504 PMC4207638

[56] Dong Y Zhang X Xie M Arefnezhad B Wang Z Wang W. Reference genome of wild goat (*Capra aegagrus*) and sequencing of goat breeds provide insight into genic basis of goat domestication. BMC Genomics. (2015) 16:431. 10.1186/s12864-015-1606-1 26044654 PMC4455334

[57] Kader A Liu X Dong K Song S Pan J Yang M. Identification of copy number variations in three chinese horse breeds using 70k single nucleotide polymorphism beadchip array. Anim Genet. (2016) 47:560–9. 10.1111/age.12451 27440410

[58] Han H Zhao X Xia X Chen H Lei C Dang R. Copy number variations of five y chromosome genes in donkeys. Arch Anim Breed. (2017) 60:391–7. 10.5194/aab-60-391-2017

[59] Ma Q Liu X Pan J Ma L Ma Y He X. Genome-wide detection of copy number variation in chinese indigenous sheep using an ovine high-density 600 k SNP array. Sci Rep. (2017) 7:912. 10.1038/s41598-017-00847-9 28424525 PMC5430420

[60] Liu M Zhou Y Rosen BD Van Tassell CP Stella A Tosser-Klopp G. Diversity of copy number variation in the worldwide goat population. Heredity. (2019) 122:636–46. 10.1038/s41437-018-0150-6 30401973 PMC6462038

[61] Al Abri MA Holl HM Kalla SE Sutter NB Brooks SA. Whole genome detection of sequence and structural polymorphism in six diverse horses. PLoS ONE. (2020) 15:e230899. 10.1371/journal.pone.0230899 32271776 PMC7144971

[62] Guan D Martínez A Castelló A Landi V Luigi-Sierra MG Fernández-Álvarez J. genome-wide analysis of copy number variation in Murciano-Granadina goats. Genetics Select Evol. (2020) 52:1–0. 10.1186/s12711-020-00564-4 32770942 PMC7414533

[63] Mei C Junjvlieke Z Raza SHA Wang H Cheng G Zhao C. Copy number variation detection in chinese indigenous cattle by whole genome sequencing. Genomics. (2020) 112:831–6. 10.1016/j.ygeno.2019.05.023 31145994

[64] He Y Hong Q Zhou D Wang S Yang B Yuan Y. Genome-wide selective detection of mile red-bone goat using next-generation sequencing technology. Ecol E11. (2021) 14805–12. 10.1002/ece3.8165 34765142 PMC8571596

[65] Nandolo W Mészáros G Wurzinger M Banda LJ Gondwe TN Mulindwa HA. Detection of copy number variants in african goats using whole genome sequence data. BMC Genomics. (2021) 22:398. 10.1186/s12864-021-07703-1 34051743 PMC8164248

[66] Salehian-Dehkordi H Xu Y Xu S Li X Luo L Liu Y. Genome-wide detection of copy number variations and their association with distinct phenotypes in the world's sheep. Front Genet. (2021) 12:670582. 10.3389/fgene.2021.670582 34093663 PMC8175073

[67] Zhou J Liu L Reynolds E Huang X Garrick D Shi Y. Discovering copy number variation in dual-purpose xinjiang brown cattle. Front Genet. (2022) 12. 10.3389/fgene.2021.747431 35222511 PMC8873982

[68] Taghizadeh S Gholizadeh M Rahimi-Mianji G Moradi MH Costilla R Moore S. Genome-wide identification of copy number variation and association with fat deposition in thin and fat-tailed sheep breeds. Sci Rep. (2022) 12:8834. 10.1038/s41598-022-12778-1 35614300 PMC9132911

[69] Kumar H Panigrahi M Saravanan KA Rajawat D Parida S Bhushan B. Genome-wide detection of copy number variations in tharparkar cattle. Anim Biotechnol. (2023) 34:448–55. 10.1080/10495398.2021.1942027 34191685

[70] Fontanesi L Martelli PL Beretti F Riggio V Dall'Olio S Colombo M. An initial comparative map of copy number variations in the goat (*Capra hircus*) genome. BMC genomics. (2010) 11:1–5. 10.1186/1471-2164-11-639 21083884 PMC3011854

[71] Feuk L Carson AR Scherer SW. Structural variation in the human genome. Nat Rev Genet. (2006) 7:85–97. 10.1038/nrg1767 16418744

[72] Mei-lin J Zeng-kui L Qing L Xiao-juan F Ming-xing C Cai-hongW. Research progress on copy number variation of livestock and poultry. J Agric Biotechnol. (2019) 10:1840–48.

[73] Fernandes AC da Silva VH Goes CP Moreira GC Godoy TF Ibelli AM. Genome-wide detection of CNVs and their association with performance traits in broilers. BMC Genomics. (2021) 22:1–8. 10.1186/s12864-021-07676-1 34001004 PMC8130382

[74] Pokrovac I Pezer Ž. Recent advances and current challenges in population genomics of structural variation in animals and plants. Front Genet. (2022) 13:1060898. 10.3389/fgene.2022.1060898 36523759 PMC9745067

[75] Hsieh P Vollger MR Dang V Porubsky D Baker C Cantsilieris S. Adaptive archaic introgression of copy number variants and the discovery of previously unknown human genes. Science. (2019) 366: aax2083. 10.1126/science.aax2083 31624180 PMC6860971

[76] Freeman JL Perry GH Feuk L Redon R McCarroll SA Altshuler DM. Copy number variation: new insights in genome diversity. Genome Res. (2006) 16:949–61. 10.1101/gr.3677206 16809666

[77] Pös O Radvanszky J Buglyó G Pös Z Rusnakova D Nagy B. copy number variation: main characteristics, evolutionary significance, and pathological aspects. Biomed J. (2021) 44:548–59. 34649833 10.1016/j.bj.2021.02.003 PMC8640565

[78] Huang Y Li Y Wang X Yu J Cai Y Zheng Z. An atlas of cnv maps in cattle, goat and sheep. Sci China Life Sci. (2021) 64:1747–64. 10.1007/s11427-020-1850-x 33486588

[79] Lupski JR Stankiewicz P. Genomic disorders: molecular mechanisms for rearrangements and conveyed phenotypes. PLoS Genet. (2005) 1:e49. 10.1371/journal.pgen.0010049 16444292 PMC1352149

[80] Gu W Zhang F Lupski JR. Mechanisms for human genomic rearrangements. Pathogenetics. (2008) 1:4. 10.1186/1755-8417-1-4 19014668 PMC2583991

[81] Lee JA Carvalho CMB Lupski JR. A dna replication mechanism for generating nonrecurrent rearrangements associated with genomic disorders. Cell. (2007) 131:1235–47. 10.1016/j.cell.2007.11.037 18160035

[82] Pinkel D Albertson DG. Comparative genomic hybridization. Annu Rev Genomics Hum Genet. (2005) 6:331–54. 10.1146/annurev.genom.6.080604.162140 16124865

[83] Chehbani F Tomaiuolo P Picinelli C Baccarin M Castronovo P Scattoni ML. Yield of array-cgh analysis in tunisian children with autism spectrum disorder. Mol Genet Genom Med. (2022) 10:e1939. 10.1002/mgg3.1939 35762097 PMC9356560

[84] Kowalczyk K Smyk M Bartnik-Głaska M Plaskota I Wiśniowiecka-Kowalnik B Bernaciak J. Application of array comparative genomic hybridization (ACGH) for identification of chromosomal aberrations in the recurrent pregnancy loss. J Assist Reprod Genet. (2022) 39:357–67. 10.1007/s10815-022-02400-8 35079943 PMC8956756

[85] Oostlander AE Meijer GA Ylstra B. Microarray-based comparative genomic hybridization and its applications in human genetics. Clin Genet. (2004) 66:488–95. 10.1111/j.1399-0004.2004.00322.x 15521975

[86] De Ravel D Devriendt TJL Fryns K Vermeesch JJR. What's new in karyotyping? The move towards array comparative genomic hybridisation (CGH). Eur J Pediatr. (2007) 166:637–43. 10.1007/s00431-007-0463-6 17372759

[87] Vissers LELM de Vries BBA Osoegawa K Janssen IM Feuth T Choy CO. Array-based comparative genomic hybridization for the genomewide detection of submicroscopic chromosomal abnormalities. The Am J Hum Genetics. (2003) 73:1261–70. 10.1086/379977 14628292 PMC1180392

[88] Weiss MM Snijders AM Kuipers EJ Ylstra B Pinkel D Meuwissen SG. Determination of amplicon boundaries at 20q13. 2 in tissue samples of human gastric adenocarcinomas by high-resolution microarray comparative genomic hybridization. The J Pathol Soc Great Britain Ireland. (2003) 200:320–6. 10.1002/path.1359 12845628

[89] LaFramboise T. Single nucleotide polymorphism arrays: a decade of biological, computational and technological advances. Nucleic Acids Res. (2009) 37:4181–93. 10.1093/nar/gkp552 19570852 PMC2715261

[90] Nowak D Hofmann W Koeffler HP. Genome-wide mapping of copy number variations using snp arrays. Transfus Med Hemother. (2009) 36:246–51. 10.1159/000225372 21049075 PMC2941829

[91] Walker BA Morgan GJ. Use of single nucleotide polymorphism–based mapping arrays to detect copy number changes and loss of heterozygosity in multiple myeloma. Clin Lymphoma Myeloma. (2006) 7:186–92. 10.3816/CLM.2006.n.057 17229333

[92] Metzger J Philipp U Lopes MS da Camara Machado A Felicetti M Silvestrelli M. Analysis of copy number variants by three detection algorithms and their association with body size in horses. BMC Genomics. (2013) 14:1–5. 10.1186/1471-2164-14-487 23865711 PMC3720552

[93] Zhao M Wang Q Wang Q Jia P Zhao Z. Computational tools for copy number variation (cnv) detection using next-generation sequencing data: features and perspectives. BMC Bioinf. (2013) 14:S1. 10.1186/1471-2105-14-S11-S1 24564169 PMC3846878

[94] Liu GE Bickhart DM. Copy number variation in the cattle genome. Funct Integr Genomics. (2012) 12:609–24. 10.1007/s10142-012-0289-9 22790923

[95] Liu L Li Y Li S Hu N He Y Pong R. Comparison of next-generation sequencing systems. J Biomed Biotechnol. (2012) 2012:251364. 10.1155/2012/251364 22829749 PMC3398667

[96] Grada A Weinbrecht K. Next-generation sequencing: methodology and application. J Invest Dermatol. (2013) 133:1–4. 10.1038/jid.2013.248 23856935

[97] Chen D Xu Y Ding C Wang Y Fu Y Cai B. The inconsistency between two major aneuploidy-screening platforms—single-nucleotide polymorphism array and next-generation sequencing—in the detection of embryo mosaicism. BMC Genomics. (2022) 23:62. 10.1186/s12864-022-08294-1 35042471 PMC8764859

[98] Bae JS Cheong HS Kim LH NamGung S Park TJ Chun JY. Identification of copy number variations and common deletion polymorphisms in cattle. BMC Genomics. (2010) 11:1–10. 10.1186/1471-2164-11-232 20377913 PMC2859865

[99] Bickhart DM Hou Y Schroeder SG Alkan C Cardone MF Matukumalli LK. Copy number variation of individual cattle genomes using next-generation sequencing. Genome Res. (2012) 22:778–90. 10.1101/gr.133967.111 22300768 PMC3317159

[100] Zhang L Jia S Yang M Xu Y Li C Sun J. Detection of copy number variations and their effects in chinese bulls. BMC Genomics. (2014) 15:480. 10.1186/1471-2164-15-480 24935859 PMC4073501

[101] Da Silva D Giachetto JM Da Silva PF Cintra LO Paiva LC Yamagishi SR. Genome-wide copy number variation (CNV) detection in nelore cattle reveals highly frequent variants in genome regions harboring QTLS affecting production traits. BMC Genomics. (2016) 17:454. 10.1186/s12864-016-2752-9 27297173 PMC4907077

[102] Zhou Y Connor EE Wiggans GR Lu Y Tempelman RJ Schroeder SG. Genome-wide copy number variant analysis reveals variants associated with 10 diverse production traits in holstein cattle. BMC Genomics. (2018) 19:314. 10.1186/s12864-018-4699-5 29716533 PMC5930521

[103] Kooverjee BB Soma P van der Nest MA Scholtz MM Neser FW. Copy number variation discovery in South African Nguni-Sired and Bonsmara-Sired crossbred cattle. Animals. (2023) 13:2513. 10.3390/ani13152513 37570321 PMC10417447

[104] Sun T Pei S Liu Y Hanif Q Xu H Chen N. Whole genome sequencing of simmental cattle for snp and cnv discovery. BMC Genomics. (2023) 24:179. 10.1186/s12864-023-09248-x 37020271 PMC10077681

[105] Liu S Kang X Catacchio CR Liu M Fang L Schroeder SG. Computational detection and experimental validation of segmental duplications and associated copy number variations in water buffalo (*Bubalus bubalis*). Funct Integr Genomics. (2019) 19:409–19. 10.1007/s10142-019-00657-4 30734132

[106] Ma Y Zhang Q Lu Z Zhao X Zhang Y. Analysis of copy number variations by snp50 beadchip array in chinese sheep. Genomics. (2015) 106:295–300. 10.1016/j.ygeno.2015.08.001 26244906

[107] Zhu C Fan H Yuan Z Hu S Ma X Xuan J. Genome-wide detection of cnvs in chinese indigenous sheep with different types of tails using ovine high-density 600k snp arrays. Sci Rep. (2016) 6:27822. 10.1038/srep27822 27282145 PMC4901276

[108] Yang L Xu L Zhou Y Liu M Wang L Kijas JW. Diversity of copy number variation in a worldwide population of sheep. Genomics. (2018) 110:143–8. 10.1016/j.ygeno.2017.09.005 28917637

[109] Kang X Li M Liu M Liu S Pan MG Wiggans GR. Copy number variation analysis reveals variants associated with milk production traits in dairy goats. Genomics. (2020) 112:4934–7. 10.1016/j.ygeno.2020.09.007 32898641

[110] Yuan C Lu Z Guo T Yue Y Wang X Wang T. A global analysis of cnvs in chinese indigenous fine-wool sheep populations using whole-genome resequencing. BMC Genomics. (2021) 22:78. 10.1186/s12864-021-07387-7 33485316 PMC7825165

[111] Igoshin AV Deniskova TE Yurchenko AA Yudin NS Dotsev AV Selionova MI. Copy number variants in genomes of local sheep breeds from russia. Anim Genet. (2022) 53:119–32. 10.1111/age.13163 34904242

[112] Salehian-Dehkordi H Huang J Pirany N Mehrban H Lv X Sun W. Genomic landscape of copy number variations and their associations with climatic variables in the worldandrsquo;s sheep. Genes. (2023) 14:1256. 10.3390/genes14061256 37372436 PMC10298528

[113] Dupuis MC Zhang Z Durkin K Charlier C Lekeux P Georges M. Detection of copy number variants in the horse genome and examination of their association with recurrent laryngeal neuropathy. Anim Genet. (2013) 44:206–8. 10.1111/j.1365-2052.2012.02373.x 22582820

[114] Wang W Wang S Hou C Xing Y Cao J Wu K. Genome-wide detection of copy number variations among diverse horse breeds by array cgh. PLoS ONE. (2014) 9:e86860. 10.1371/journal.pone.0086860 24497987 PMC3907382

[115] Schurink A Da Silva D Velie VH Dibbits BD Crooijmans BW. Copy number variations in friesian horses and genetic risk factors for insect bite hypersensitivity. BMC Genet. (2018) 19:49. 10.1186/s12863-018-0657-0 30060732 PMC6065148

[116] Solé M Ablondi M Binzer-Panchal A Velie BD Hollfelder N Buys N. Inter-and intra-breed genome-wide copy number diversity in a large cohort of European equine breeds. BMC Genomics. (2019) 20:1–2. 10.1186/s12864-019-6141-z 31640551 PMC6805398

[117] Wang C Li H Guo Y Huang J Sun Y Min J. Donkey genomes provide new insights into domestication and selection for coat color. Nat Commun. (2020) 11:6014. 10.1038/s41467-020-19813-7 33293529 PMC7723042

[118] Huang Y Xu T Li L Li Z. Jun-yong W, Hong C, Zhao LC. Research progress of copy number variation of cattle and sheep china cattle. Science. (2018) 44:55–60. 10.3969/j.issn.1001-9111.2018.04.015

[119] Keel BN Lindholm-Perry AK Snelling WM. Evolutionary and functional features of copy number variation in the cattle genome. Front Genet. (2016) 7:207. 10.3389/fgene.2016.00207 27920798 PMC5118444

[120] Corbi-Botto CM Morales-Durand H Zappa ME Sadaba SA Peral-García P Giovambattista G. Genomic structural diversity in criollo argentino horses: analysis of copy number variations. Gene. (2019) 695:26–31. 10.1016/j.gene.2018.12.067 30763671

[121] Nozawa M Kawahara Y Nei M. Genomic drift and copy number variation of sensory receptor genes in humans. Proc Nat Acad Sci. (2007) 104:20421–6. 10.1073/pnas.0709956104 18077390 PMC2154446

[122] Young JM Endicott RM Parghi SS Walker M Kidd JM Trask BJ. Extensive copy-number variation of the human olfactory receptor gene family. The Am J Hum Genetics. (2008) 83:228–42. 10.1016/j.ajhg.2008.07.005 18674749 PMC2495065

[123] Veerappa AM Vishweswaraiah S Lingaiah K Murthy M Manjegowda DS Nayaka R. Unravelling the complexity of human olfactory receptor repertoire by copy number analysis across population using high resolution arrays. PLoS ONE. (2013) 8:e66843. 10.1371/journal.pone.0066843 23843967 PMC3700933

[124] Spehr M Munger SD. Olfactory receptors: g protein-coupled receptors and beyond. J Neurochem. (2009) 109:1570–83. 10.1111/j.1471-4159.2009.06085.x 19383089 PMC4455932

[125] Palouzier-Paulignan B Lacroix MC Aime P Baly C Caillol M Congar P. Olfaction under metabolic influences. Chem Senses. (2012) 37:769–97. 10.1093/chemse/bjs059 22832483 PMC3529618

[126] Ghosh S Das PJ McQueen CM Gerber V Swiderski CE Lavoie JP. Analysis of genomic copy number variation in equine recurrent airway obstruction (heaves). Anim Genet. (2016) 47:334–44. 10.1111/age.12426 26932307

[127] Zhang F Gu W Hurles ME Lupski JR. Copy number variation in human health, disease, and evolution. Annu Rev Genomics Hum Genet. (2009) 10:451–81. 10.1146/annurev.genom.9.081307.164217 19715442 PMC4472309

[128] Shi T Xu Y Yang M Huang Y Lan X Lei C. Copy number variations at lepr gene locus associated with gene expression and phenotypic traits in chinese cattle. Anim Sci J. (2016) 87:336–43. 10.1111/asj.12531 26568073

[129] Xu L Hou Y Bickhart DM Song J Van Tassell CP Sonstegard TS. genome-wide survey reveals a deletion polymorphism associated with resistance to gastrointestinal nematodes in Angus cattle. Funct Integ Genomics. (2014) 14:333–9. 10.1007/s10142-014-0371-6 24718732

[130] Liu M Li B Huang Y Yang M Lan X Lei C. Copy number variation of bovine MAPK10 modulates the transcriptional activity and affects growth traits. Livest Sci. (2016) 194:44–50. 10.1016/j.livsci.2016.09.014

[131] Yang M Lv J Zhang L Li M Zhou Y Lan X. Association study and expression analysis of cyp4a11 gene copy number variation in chinese cattle. Sci Rep. (2017) 7:46599. 10.1038/srep46599 28492277 PMC5425913

[132] Zhang GM Zheng L He H Song CC Zhang ZJ Cao XK. Associations of GBP2 gene copy number variations with growth traits and transcriptional expression in Chinese cattle. Gene. (2018) 647:101–6. 10.1016/j.gene.2018.01.004 29325733

[133] Ma YL Wen YF Cao XK Cheng J Huang YZ Ma Y. Copy number variation (CNV) in the IGF1R gene across four cattle breeds and its association with economic traits. Archives Anim Breeding. (2019) 62:171–9. 10.5194/aab-62-171-2019 31807627 PMC6852844

[134] Xu JW Zheng L Li LJ Yao YF Hua H Yang SZ. Novel copy number variation of the KLF3 gene is associated with growth traits in beef cattle. Gene. (2019) 680:99–104. 10.1016/j.gene.2018.08.040 30099021

[135] Zheng L Xu JW Li JC Wang DH An QM Xu LN. Distribution and association study in copy number variation of KCNJ12 gene across four Chinese cattle populations. Gene. (2019) 689:90–6. 10.1016/j.gene.2018.12.019 30572095

[136] Yang P Zhang Z Xu J Qu K Lyv S Wang X. The association of the copy number variation of the MLLT10 gene with growth traits of Chinese cattle. Animals. (2020) 10:250. 10.3390/ani10020250 32033330 PMC7070264

[137] Hao D Wang X Thomsen B Kadarmideen HN Wang X Lan X. Copy number variations and expression levels of guanylate-binding protein 6 gene associated with growth traits of Chinese cattle. Animals. (2020) 10:566. 10.3390/ani10040566 32230930 PMC7222342

[138] Tang J Shen X Yang Y Yang H Qi A Yang S. Two different copy number variations of the clcn2 gene in chinese cattle and their association with growth traits. Animals. (2021) 12:41. 10.3390/ani12010041 35011147 PMC8749635

[139] Hu L Yu J Huang R Yang P Zhang Z Chai Y. Copy number variation of the CCDC39 gene is associated with growth traits in Chinese cattle. Vet Med Sci. (2022) 8:917–24. 10.1002/vms3.712 35233959 PMC8959325

[140] Yang P Cai C Niu M Liu X Wang H Liang H. of copy number variation of PLA2G2A gene to growth traits in Chinese cattle. Gene. (2022) 809:146014. 10.1016/j.gene.2021.146014 34655722

[141] Yang H Yue B Yang Y Tang J Yang S Qi A. Distribution of copy number variation in syt11 gene and its association with growth conformation traits in Chinese cattle. Biology. (2022) 11:223. 10.3390/biology11020223 35205089 PMC8869484

[142] Yao Z Li J Zhang Z Chai Y Liu X Li J. The relationship between MFN1 copy number variation and growth traits of beef cattle. Gene. (2022) 811:146071. 10.1016/j.gene.2021.146071 34864096

[143] Li X Ding X Liu L Yang P Yao Z Lei C. Copy number variation of bovine DYNC1I2 gene is associated with body conformation traits in chinese beef cattle. Gene. (2022) 810:146060. 10.1016/j.gene.2021.146060 34740731

[144] Zhang J Zhang Z Liu X Chai Y Yang P Li J. number variation of WBP1L gene revealed its association with growth traits across chinese cattle populations. J Agric Sci. (2022) 160:528–34. 10.1017/S002185962200038732126252

[145] Chen Y Peng W Zhang Z Liu X Yang P Fu C. The relationship between MUC19 copy number variation and growth traits of Chinese cattle. Gene. (2023) 851:147010. 10.1016/j.gene.2022.147010 36349576

[146] Liang J Liu X Yang P Yao Z Qu K Wang H. Copy number variation of gal3st1 gene is associated with growth traits of chinese cattle. Anim Biotechnol. (2023) 34:672–8. 10.1080/10495398.2021.1996385 35001788

[147] Liu X Yang P Sun H Zhang Z Cai C Xu J. analysis of VAMP7 gene reveals variation associated with growth traits in Chinese cattle. Anim Biotechnol. (2023) 34:1095–101. 10.1080/10495398.2021.2011741 35236249

[148] Song X Li X Liu X Zhang Z Ding X Chai Y. Copy number variation of the ZNF679 gene in cattle and its association analysis with growth traits. Anim Biotechnol. (2023) 21:1–7. 10.1080/10495398.2023.2185628 37093180

[149] Jiang R Cheng J Cao XK Ma YL Chaogetu B Huang YZ. Copy number variation of the SHE gene in sheep and its association with economic traits. Animals. (2019) 9:531. 10.3390/ani9080531 31390723 PMC6720781

[150] Wang X Cao X Wen Y Ma Y Elnour IE Huang Y. Associations of ORMDL1 gene copy number variations with growth traits in four Chinese sheep breeds. Arch Anim Breeding. (2019) 62:571–8. 10.5194/aab-62-571-2019 31807669 PMC6853131

[151] Cheng J Jiang R Yang Y Cao X Huang Y Lan X. Association analysis of KMT2D copy number variation as a positional candidate for growth traits. Gene. (2020) 753:144799. 10.1016/j.gene.2020.144799 32446916

[152] Toremurat Z Ibrahim EE Huang YZ Lan X Pi L Chaogetu B. Copy number variations of TOP2B gene are associated with growth traits in Chinese sheep breeds. Anim Biotechnol. (2022) 33:85–9. 10.1080/10495398.2020.1773490 32498592

[153] Yang Z Cao X Ma Y Cheng J Song C Jiang R. Novel copy number variation of the bag4 gene is associated with growth traits in three chinese sheep populations. Anim Biotechnol. (2021) 32:461–9. 10.1080/10495398.2020.1719124 32022644

[154] Wen Y Wang E Wang X Qing S Chaogetu B Wang C. Copy number variations of LRRFIP1 gene and the relationship with growth traits in four Chinese sheep. Anim Biotechnol. (2023) 34:3008–15. 10.1080/10495398.2022.2126981 36170043

[155] Wang X Wang Y Cao X Huang Y Li P Lan X. Copy number variations of the kat6a gene are associated with body measurements of chinese sheep breeds. Anim Biotechnol. (2023) 34:947–54. 10.1080/10495398.2021.2005616 34842492

[156] Feng Z Li X Cheng J Jiang R Huang R Wang D. Copy number variation of the pigy gene in sheep and its association analysis with growth traits. Animals. (2020) 10:688. 10.3390/ani10040688 32326606 PMC7222781

[157] Shi SY Li LJ Zhang ZJ Wang EY Wang J Xu JW. Copy number variation of MYLK4 gene and its growth traits of *Capra hircus* (goat). Anim Biotechnol. (2020) 31:532–7. 10.1080/10495398.2019.1635137 31280665

[158] Li L Yang P Shi S Zhang Z Shi Q Xu J. Association analysis to copy number variation (CNV) of Opn4 gene with growth traits of goats. Animals. (2020) 10:441. 10.3390/ani10030441 32155759 PMC7143651

[159] Liu M Woodward-Greene J Kang X Pan MG Rosen B Van Tassell CP. Genome-wide CNV analysis revealed variants associated with growth traits in African indigenous goats. Genomics. (2020) 112:1477–80. 10.1016/j.ygeno.2019.08.018 31450006

[160] Xu Z Wang X Zhang Z An Q Wen Y Wang D. Copy number variation of CADM2 gene revealed its association with growth traits across Chinese *Capra hircus* (goat) populations. Gene. (2020) 741:144519. 10.1016/j.gene.2020.144519 32126252

[161] Wang Q Wei Z Zhu H Pan C Akhatayeva Z Song X. Goat pleomorphic adenoma gene 1 (PLAG1): mRNA expression, CNV detection and associations with growth traits. Animals. (2023) 13:2023. 10.3390/ani13122023 37370533 PMC10294908

[162] Song X Bai Y Yuan R Zhu H Lan X Qu L. InDel and CNV within the AKAP13 gene revealing strong associations with growth traits in goat. Animals. (2023) 13:2746. 10.3390/ani13172746 37685010 PMC10487263

[163] Wang Q Song X Bi Y Zhu H Wu X Guo Z. Detection distribution of CNVs of SNX29 in three goat breeds and their associations with growth traits. Front Vet Sci. (2023) 10:1132833. 10.3389/fvets.2023.1132833 37706075 PMC10495836

[164] Pei SW Qin F Li WH Li FD Yue XP. Copy number variation of ZNF280AY across 21 cattle breeds and its association with the reproductive traits of Holstein and Simmental bulls. J Dairy Sci. (2019) 102:7226–36. 10.3168/jds.2018-16063 31202648

[165] Yue XP Dechow C Chang TC DeJarnette JM Marshall CE Lei CZ. Copy number variations of the extensively amplified Y-linked genes, HSFY and ZNF280BY, in cattle and their association with male reproductive traits in Holstein bulls. BMC Genomics. (2014) 15:1–2. 10.1186/1471-2164-15-113 24507556 PMC3924399

[166] Pei S Xu H Wang L Li F Li W Yue X. Copy number variation of ZNF280BY across eight sheep breeds and its association with testicular size of Hu sheep. J Anim Sci. (2022) 100:skac232. 10.1093/jas/skac232 35775620 PMC9467034

[167] Wang Q Bi Y Wang Z Zhu H Liu M Wu X. Goat SNX29: mRNA expression, InDel and CNV detection, and their associations with litter size. Front Vet Sci. (2022) 9:981315. 10.3389/fvets.2022.981315 36032302 PMC9399746

[168] Bai Y Zhang T Liu N Wang C Guo Z Pan C. Investigation of copy number variations (CNVs) of the goat PPP3CA gene and their effect on litter size and semen quality. Animals. (2022) 12:445. 10.3390/ani12040445 35203154 PMC8868321

[169] Zhang R Wang J Zhang T Zhai H Shen W. Copy-number variation in goat genome sequence: a comparative analysis of the different litter size trait groups. Gene. (2019) 696:40–6. 10.1016/j.gene.2019.02.027 30772519

[170] Janečka JE Davis BW Ghosh S Paria N Das PJ Orlando L. Horse y chromosome assembly displays unique evolutionary features and putative stallion fertility genes. Nat Commun. (2018) 9:2945. 10.1038/s41467-018-05290-6 30054462 PMC6063916

[171] Trigo BB Utsunomiya ATH Fortunato AAAD Milanesi M Torrecilha RBP Lamb H. Variants at the asip locus contribute to coat color darkening in nellore cattle. Genet Sel E. (2021) 53:40. 10.1186/s12711-021-00633-2 33910501 PMC8082809

[172] Liang D Zhao P Si J Fang L Pairo-Castineira E Hu X. Genomic analysis revealed a convergent evolution of line-1 in coat color: a case study in water buffaloes (bubalus bubalis). Mol Biol E38. (2021) 1122–36. 10.1093/molbev/msaa279 33212507 PMC7947781

[173] Fontanesi L Beretti F Riggio V Gómez González E Dall Olio S Davoli R. Copy number variation and missense mutations of the agouti signaling protein (asip) gene in goat breeds with different coat colors. Cytogenet Genome Res. (2010) 126:333–47. 10.1159/000268089 20016133

[174] Menzi F Keller I Reber I Beck J Brenig B Schütz E. Genomic amplification of the caprine ednra locus might lead to a dose dependent loss of pigmentation. Sci Rep. (2016) 6:28438. 10.1038/srep28438 27329507 PMC4916431

[175] Guo J Sun X Mao A Liu H Zhan S Li L. 13 42-kb tandem duplication at the ASIP locus is strongly associated with the depigmentation phenotype of non-classic Swiss markings in goats. BMC Genomics. (2022) 23:437. 10.1186/s12864-022-08672-9 35698044 PMC9190080

[176] Rosengren Pielberg G Golovko A Sundström E Curik I Lennartsson J Seltenhammer MH. A cis-acting regulatory mutation causes premature hair graying and susceptibility to melanoma in the horse. Nature Genet. (2008) 40:1004–9. 10.1038/ng.185 18641652

[177] Drögemüller C Distl O Leeb T. Partial deletion of the bovine ed1 gene causes anhidrotic ectodermal dysplasia in cattle. Genome Res. (2001) 11:1699–705. 10.1101/gr.182501 11591646 PMC311120

[178] Guang-Xin E Yang BG Zhu YB Duang XH Basang WD Luo XL. Genome-wide selective sweep analysis of the high-altitude adaptability of yaks by using the copy number variant. Biotech. (2020) 10:1–6. 10.1007/s13205-020-02254-w 32432020 PMC7235113

[179] Guo S Wu X Pei J Wang X Bao P Xiong L. Genome-wide cnv analysis reveals variants associated with high-altitude adaptation and meat traits in qaidam cattle. Electron J Biotechnol. (2021) 54:8–16. 10.1016/j.ejbt.2021.07.006

[180] Huang Y Shi QT Shi S Yang P Zhang Z Lyu S. Association between copy number variation of serpina3-1 gene and growth traits in chinese cattle. Anim Biotechnol. (2023) 34:1524–31. 10.1080/10495398.2022.2038183 35209806

[181] Durkin K Coppieters W Drögemüller C Ahariz N Cambisano N Druet T. Serial translocation by means of circular intermediates underlies colour sidedness in cattle. Nature. (2012) 482:81–4. 10.1038/nature10757 22297974

[182] Durán Aguilar M Román Ponce SI Ruiz López FJ González Padilla E Vásquez Peláez CG Bagnato A. Genome-wide association study for milk somatic cell score in holstein cattle using copy number variation as markers. J Anim Breeding Genetics. (2017) 134:49–59. 10.1111/jbg.12238 27578198

